# Enhancing occluded and standard bird object recognition using fuzzy-based ensembled computer vision approach with convolutional neural network

**DOI:** 10.1038/s41598-025-05465-4

**Published:** 2025-07-01

**Authors:** L. Richard, G. H. Dhruthi, M. Ashwin Kumar, Anoushka Ghosh, R. Arumuga Arun, N. Priyanka

**Affiliations:** School of Computer Science and Engineering, Vellore Institute of Technology, Vellore, Tamil Nadu India

**Keywords:** Bird species classification, Deep learning, Convolutional neural networks, Ensemble learning, Fuzzy logic, Computational science, Computer science

## Abstract

Classifying bird species is essential for ecological study and biodiversity protection, currently, conventional approaches are frequently laborious and susceptible to mistakes. Convolutional Neural Networks (CNNs) provide a more reliable option for feature extraction and classification. By combining the top three independently superior CNN architectures for recognizing bird species from the DenseNet and ResNet families into a fuzzy-based ensemble learning framework, this study helps increase classification accuracy, especially for occluded bird objects. The model improves generalization by using 11,352 images collected from the Caltech-UCSD Birds-200-2011 and Birds525 Species-Image Classification datasets, as well as sophisticated augmentation approaches. Our ensemble method adaptively allocates model weights based on feature contributions found using fuzzy logic, in contrast to existing methods that have trouble with obstructed images. Since, every CNN model candidate in the suggested fuzzy-based ensemble learning showed excellent classification performance, the proposed fuzzy-based ensemble approach achieving 98.73% accuracy, a 98.75% F1-score for standard images, and 95.78% accuracy, and a 95.1% F1-score for occluded images, the results indicating performance improvements of 2% for standard and 9% for occluded bird images over the methods used in existing research work. Furthermore, as compared to the individual CNN candidates in the proposed fuzzy-based ensemble, this indicates a 2–5% performance improvement for standard bird images and a 4–7% performance improvement for occluded bird images. Additionally, the reliability and significance of the observed performance increases are verified by statistical validation of the results using p-value and F-statistic testing and 95% Confidence Intervals.

## Introduction

Birds are crucial for maintaining ecological balance through seed dispersal, pollination, and pest control, predominant for biodiversity and a healthy ecosystem^1^. The accurate identification and classification of bird species are important for ecological research and biodiversity conservation, primarily in monitoring environmental changes^2^. Traditional methods of classification based on morphological analysis are limited, time-consuming, and prone to observer subjectivity. However, advancements in digital imaging and high-intensity image recognition techniques provide robust solutions for automatic bird classification. With the help of these studies, we can accurately collect, analyse, and monitor with high precision^3^.

As a response to these needs, deep-learning techniques have opened new dimensions of automation and precision in the detailed recognition of bird species. For instance, Convolutional Neural Networks (CNNs) have been highly effective in handling large datasets of images where there is automatic or heavy reduction dependency on manual extraction of the features^4,5^. This automation in feature extraction means that CNNs can automatically detect subtle characteristics like plumage patterns, feather coloration, beak structure, or body shape. All of these features are very important in identifying different kinds of bird species. CNNs help simplify the classification process, which results in faster and more accurate results, furthering the reliability of biodiversity studies and conservation^5^.

Another practical approach for bird species classification, especially useful when large labeled datasets of bird images are scarce, is transfer learning. Transfer learning utilizes a pre-trained model as a foundation for learning a more complex classification task than it was originally trained on. This allows the model to learn faster and with greater accuracy^6,7^. The methodology enables fine-tuning models specific for a given species of bird, according to prior knowledge, and augmenting accuracy if the number of data is limited, which makes it highly suitable for ecological research^7^.

The ability of CNNs to infer a spatial hierarchy from image data led to exceptional performance in bird classification tasks, achieving a better hit rate than traditional methods. Their multilayer architectures make the detection of complex visual features very hard if interpreted manually. These are color and textural patterns, beak and wing shapes, and forms of bodies, where the scope of CNNs is suitably achieved to tackle the task of classification to a high accuracy level^8^. The ability of CNNs to model complex features in an image not only optimizes their task of classification but also works to fulfill the increasing requests for systematic inputs into ecological research and biodiversity initiatives^8^.

In addition to single CNN models, integrating multiple models within a single framework or using multi-CNN architectures can leverage the best features of different neural network structures. As a result, the models become more robust even across different datasets and settings and often within huge collections of images, resulting in a greater degree of classification accuracy. Hybrid learning approaches fused with traditional machine learning or neural network (NN) models deliver the utmost level of performance in bird species classification^2^. Hybrid models have enhanced generalization and robustness and are thus crucial in tasks of fine distinctions among species with subtle visual differences. Hybrid approaches are particularly useful in the subtle world of bird classification, where detecting subtle but important differences can significantly influence accuracy and reliability in both research and conservation-based applications^8^.

Ensemble learning combines predictions from multiple models to enhance accuracy and reliability, especially in cases like partially hidden birds, where individual models may fail^9^. Our work has further been able to classify images of partially occluded or hidden birds, thus showing its ability to work well in challenging situations. Such situations of occlusions occur generally when birds are partially hidden from foliage, branches, or various environmental elements. However, few of the studies have dealt with this challenge, and consequently, there is a gap in handling such real-life scenarios. Furthermore, we use a fuzzy-based ensemble learning technique that improves classification resilience by adaptively assigning models the best weights according to their contributions. In occluded environments, where there is considerable uncertainty about how features are represented, our approach enables improved accuracy and refines decision bounds.

Fuzzy-based ensemble learning dynamically adjusts the model weights according to their feature-level contributions, unlike conventional ensemble approaches that frequently depend on static or heuristically determined weight assignments for individual models. This adaptation becomes especially beneficial in real-world ecological contexts, where bird images frequently suffer from occlusions, noise, or uneven lighting. Fuzzy logic is used by the ensemble process to efficiently control imprecision and uncertainty in the extracted features, producing predictions that are more reliable and accurate. Because of this, our approach ensures more reliable bird identification, minimizes data loss, and improves ecological research outcomes by focusing on occluded images. In addition, the model classifies with high confidence scores, which further enhances the accuracy and dependability of the predictions made by our model^9^. The major contributions of this work are summarized as follows:

- The study combined two distinct bird image datasets—Birds525 Species-Image Classification and Caltech-UCSD Birds-200-2011—resulting in a diverse and heterogeneous dataset. This fusion enhanced the generalization capability of the models by exposing them to varied image qualities, backgrounds, and poses, thereby increasing robustness against overfitting and improving real-world applicability.
- We conducted an extensive evaluation of several state-of-the-art pre-trained CNN architectures, utilizing transfer learning to converge the model faster and reduce computational overhead. Out of which, three were identified as top-performing models on both standard and occluded datasets.
- A hybrid learning strategy was developed by extracting feature maps from the three top-performing CNN models (DenseNet121, DenseNet169, and ResNet101v2) and feeding them into classical machine learning classifiers—Random Forest, SVM, and KNN. This approach improved classification accuracy on challenging datasets, particularly occluded images, by leveraging diverse and complementary feature representations.
- An advanced fuzzy logic-driven ensemble learning technique was proposed to address the challenge of assigning optimal contribution weights to individual models, which may perform differently across image types.
- Leveraging model confidence scores and fuzzy logic, the method dynamically adjusts weights based on performance, leading to improved adaptability and accuracy. This approach outperformed both standalone models, hybrid approaches and static ensemble strategies, achieving higher accuracy on both standard and occluded datasets.
- To further evaluate the robustness of the ensemble learning techniques under diverse and realistic ecological scenarios, we developed four challenged datasets where, two datasets simulate varying levels of visual degradation by injecting noise, another introduces realistic occlusions using artificial objects such as leaves, shadows, and branches to mimic natural environmental interference, the fourth dataset serves as an external validation set, comprising entirely unseen bird images sourced from the internet, thereby testing the generalization capability of the models on out-of-distribution data.
- By addressing partial occlusion and feature uncertainty using fuzzy ensemble logic, the proposed method ensures high accuracy and reliability in real-world bird monitoring systems, facilitating biodiversity conservation and ecological surveillance.

## Literature survey

This work’s literature review is organized into two major categories. The first examines current techniques for classifying bird species to highlight the shortcomings and difficulties found in earlier research. The second looks at proven strategies that have been applied in other contexts to identify efficient techniques that can be modified to improve the categorization performance of bird species in this study.

### Existing works on bird species classification

Recent breakthroughs in bird classification rely on deep learning models, particularly CNN architectures, to improve upon a higher classification quality and efficiency. Song^10^ reported in 2024 an enhanced ResNet-152, a much more accurate classification of birds on the Birds525 Species Image dataset with precision, recall, and F1 scores of 96.5% at 98.0%, 94.6%, and 94.7%. The benefits introduced by the ResNet architecture neatly solved the vanishing gradients problem caused by deep layers, thus improving the classification results for large datasets. However, there is a limitation in addressing the classification of occluded images, which might affect the performance when the image is partially obstructed.

Cai (2023)^11^ used the model EfficientNetB0 via transfer learning and data augmentation, and it had an accuracy of 86.7% on the Birds525 Species Image dataset of 525 species of birds. The study was evaluated in realizing that though transfer learning combined with data augmentation promotes better generalization over unseen data, certain species like the Abbots booby class remain more challenging for classification purposes due to some subtle visual differences.

Farman et al. (2023)^12^ tested CNNs with skip connections combined with VGG16 by classifying the species of birds. The authors have considered a 20-species subset, and the full dataset comprises 525 species. Accuracy achieved for 20 species by CNN was significantly high at 92%, while the performance of VGG16 remains at 0.60 accuracy while scaled to 525 species. This study underlines the inadequacy of traditional CNNs such as VGG16 to deal with large, diverse datasets, with certain architectural modifications to learn features over many species.

Wang et al. (2023)^13^ present a lightweight bird classification model based on the ShuffleNetV2 architecture specially designed for fine-grained classification using attention mechanisms and distillation knowledge. It was tested on the CUB-200-2011 dataset with F1, precision, and recall scores close to 84% as the method reached an accuracy of 87.02%. This reduces model complexity and makes it a good candidate for deployment on mobile or edge devices, avoiding the computational limits of deep CNNs and focusing on distinguishing features and knowledge distillation.

Kondaveeti et al. (2023)^14^ proposed a bird species identification network using MobileNetV2 with an average accuracy of about 84.5%. MobileNetV2 is a variant of lightweight CNN models that can be used in mobile devices and edge devices. Since it supports high-accuracy performance with low computational requirements, it attracts minimal support that can be used for computation-scarce environments, showing a balance between efficiency and performance in bird species identification applications. Nevertheless, its strength is confined to settings requiring unusually fine resolutions, a task that larger models could do more efficiently.

Yi et al. (2023)^15^ proposed an improved YOLOv5 model for fine-grained bird classification that achieved a test accuracy of 92% on a large dataset of bird species images. By modifying the standard architecture, the model could handle fine-grained classification tasks effectively, distinguishing between species with subtle visual differences. This model has its usability severely compromised with species where there is great intra-species variation in the physical features, thus making it not very viable for real-life applications in the field.

Yu and Wang^16^ (2023) proposed a Hybrid Granularities Transformer, which reached 89.4% accuracy in the fine-grained image recognition experiment. It leverages multi-granularity feature extraction with global and local details in the captured information, illustrating the potential of transformers in solving complex bird classification tasks. The approach is very computationally intensive, which can negatively affect the usability of this model in real-time or low-power applications.

Vo et al. (2023)^17^ coupled YOLOv5 with multiple CNN architectures, namely VGG19, Inception V3, and EfficientNetB3, to design a hybrid model for the detection and classification of birds. Among these, EfficientNetB3 recorded the maximum test accuracy of 98%, whereas YOLOv5 with VGG19 attained 95%, and it varied between 92% and 93% with InceptionV3. The methodology presented demonstrates the effectiveness of adopting hybrid architectures for object detection. YOLOv5 has achieved spectacular localization capabilities, while CNN models are capable of fine-grained species classification. This confirms the capability of such hybrid models in multitask scenarios involving both detection and classification. While the paper has impressive results, it does not classify occluded images, as in some real-time cases, only a partial image is captured due to obstruction.

Ngo et al. (2023)^18^ presented a bird species recognition system using an ensemble of fine-tuned CNN models with an accuracy of 90.2%. The ensemble method enhances the identification of bird species’ morphological characteristics by utilizing multiple pre-trained networks, aiming for greater accuracy in distinguishing finely graded intra-species variations. The paper discusses the advantages of fine-tuning but does not discuss the limitations of fine-tuning, like overfitting when the dataset is small or images of birds are occluded.

Kim et al. (2025)^19^ enhanced deep learning model accuracy for Amazon parrot species classification by proposing a hierarchical classification method based on transfer learning. Their hierarchical model performed better than a non-hierarchical model with a mean Average Precision (mAP) of 0.944 through the construction of a hierarchy from diagnostic morphological features. When dealing with visually similar bird categories and limited data, this hierarchical transfer learning method can be effective in classifying bird species. However, when there is an introduction of new species or physical distinctions are minimal, scalability can be compromised because it relies on carefully designed hierarchies. The literature survey of existing bird species classification is summarized in Table 1.

**Table 1:** Qualitative comparison with existing classification models.

Reference	Year	Key contribution	Merit	Demerit
^1^	2024	Introduced an enhanced ResNet- 152 model for bird classification, attaining a high accuracy on the BIRDS 525 dataset	Improves classification by addressing the vanishing gradients issue caused by residual layers	Exhibits difficulties with occluded images, which impact performance when bird parts are obscured
^2^	2023	Utilized EfficientNetB0 with data augmentation and transfer learning for classification	Attained an accuracy of 86.7%, illustrating the effectiveness of transfer learning	Challenging to classify certain species due to some subtle visual differences
^3^	2023	Used CNNs with skip connections plus VGG16 for classification	Achieved high accuracy (92%) for 20 species of South Indian bird species, highlighting the effectiveness of CNN	Performance dropped significantly for 525 species, showing the incapacity of conventional CNNs to handle extensive datasets
^4^	2023	Constructed a lightweight attention- mechanism-based bird categorization model based on ShuffleNetV2	Low processing complexity and high accuracy (87.02%) make it appropriate for mobile devices	Performance for fine-grained classification is limited by lower accuracy when compared to deeper CNNs
^5^	2023	Bird species were classified using MobileNetV2 with an accuracy of 84.5%	Due to its minimal computing requirements, it is effective for mobile and edge devices	Faces challenges classifying data at high resolution when larger models outperform smaller ones
^6^	2023	Enhanced YOLOv5 for fine-grained bird classification, achieving 92% accuracy	Successfully manages fine- grained classification by altering the conventional YOLOv5 architecture	Challenges with species that exhibit significant intraspecies variation, which restricts practical use
^7^	2023	Provided a Hybrid Granularities Transformer for fine-grained classification	Enhances challenging categorization tasks by extracting both local and global traits	Computationally demanding, which makes low-power or real-time applications challenging
^8^	2023	YOLOv5 and CNNs (VGG19, Inception V3, EfficientNetB3) are combined in this hybrid model to recognize and classify birds	Attained high test accuracy (98% with EfficientNetB3), proving hybrid architectures’ effectiveness	Struggles with occluded images, where only partial bird images are visible
^9^	2023	Proposed an ensemble of fine- tuned CNN models for bird classification	Improved accuracy with the use of several pre-trained networks	It overcomes occluded images and overfitting issues in small datasets
^10^	2023	Presents an ensemble learning with DCNNs for multi-modal image fusion	Integrates image data and features with the help of ensemble learning, improving the accuracy of species differentiation	There is little discussion of direct application to the taxonomy of birds
^11^	2021	Utilized deep learning ensembles for multimodal remote sensing image classification	Highlights the potential of ensembles for improving classification in diverse environments	Does not focus on addressing the computational complexity of the task and data imbalance
^19^	2025	Proposed a transfer learning-based hierarchical classification system for Amazon parrot species	Achieved high accuracy (mAP 0.944); useful for visually similar species with insufficient data	Scalability is limited when introducing new species or when there aren’t many physical differences
^20^	2024	Developed SFSCF- Net for FGVC by fusing cross- feature fusion with saliency suppression	Enhanced feature focus and robust semantic representation for fine-grained bird classification	High computational complexity, which precludes its use in real-time or low- resource settings

### Leveraging established methods to optimize classification performance

Sureshkumar et al. (2021)^21^ suggested a hybrid optimization-based feature selection technique for the classification of thyroid disease based on a Rough Type-2 Fuzzy SVM (RT2FSVM). By combining the firefly algorithm (FA) and butterfly optimization algorithm (BOA), their HFBO-RT2FSVM model attained 99.28% accuracy with high sensitivity and specificity compared to conventional methods. This hybrid technique could be exploited in bird image classification by choosing crucial visual features, although dependency upon tuning optimization parameters can affect stability across datasets.

Yang et al. (2024)^20^ introduced SFSCF-Net, which integrated important feature suppression and cross-feature fusion to enhance fine-grained visual categorization (FGVC). It applies a cross-feature fusion approach (CFM) for enhanced semantic representation, an object-level image generator (OIG) to suppress background noise, and a saliency feature suppression module (SFSM) to emphasize subtle features. Its sophisticated architecture, however, can cause increased computational overhead, making it less suitable for resource-limited deployments.

Joshi et al. (2021)^22^ introduced a deep learning-based ensemble for multimodal remote sensing image classification with feature extraction algorithms including VGG19, Capsule Network, and MobileNet, optimization techniques such as HCO and SSA, with a focus on achieving better results for classification problems but does not focus on addressing the computational complexity of the task and data imbalance. In the process of bird species classification, it is possible to apply a similar ensemble approach using multiple models integrated with image data to enhance the differentiation accuracy of such species in their diverse habitats.

Maseleno et al. (2023)^23^ suggested an ensemble learning approach in the fusion of multi-modal medical images using Deep Convolutional Neural Networks (DCNNs) that has improved diagnosis through the amalgamation of the different modalities’ information. This method utilizes DCNN models independently to extract specific modality features, which are then combined through a fusion module to produce a high-quality fused image. In the case of bird species classification, this multi-modal approach could be used to integrate image data and features, using ensemble learning to improve the accuracy of species differentiation. This would help gain a better understanding of the species by integrating different models.

Hasan et al. (2024)^24^ introduced an optimized weighted average ensemble-based light deep learning model integrating CNN feature extraction with machine learning classifiers to forecast cardiovascular disease from ECG images, with 99.29% accuracy in four classes. Their optimized weighted average ensemble method could be used for building an efficient bird species classification. Himel et al. (2023)^25^ suggested ensemble learning for sheep breed classification using the integration of CNN models such as Xception, VGG16, InceptionV3, InceptionResNetV2, and DenseNet121 with the application of transfer learning for increased accuracy. The technique can be applied to bird species classification by detecting subtle variations, but employing several heavy models could raise computational expenses and limit resource-constrained or real-time usage. Olisah et al. (2024)^26^ proposed a multi-input CNN ensemble classifier (MCE) based on VGG16 and stack generalization ensemble (SGE) to identify blackberry ripeness through hyperspectral imaging, with 90.2% in-field and 95.1% unseen data accuracy.

Nguyen et al. (2025) suggest a credal ensembling technique, hoping to better estimate uncertainty and make decisions by pooling probabilistic ensemble outputs^27^. Though useful for processing inconclusive images, its expensive computational requirement can restrict its application to big data sets. Jenul et al. (2022), meanwhile, introduce UBayFS, a Bayesian ensemble feature selection method that combines data-driven and expert knowledge^28^. This combination may enhance classification performance through the use of visual and expert features, although its dependency on domain expertise might influence its ability to generalize across various datasets. In addition, Jeong et al. (2024) proposed WSE-Net, a Weak Saliency Ensemble Network for person re-identification from infrared pictures^29^. By employing channel reduction (CRF), patch-based input, and grouped convolution ensembles (GCE-Net), WSE-Net obtains excellent rank-1 and mAP performance on the DBPerson-Recog-DB1 and SYSU-MM01 datasets. Overall, these works illustrate the promise of ensemble-based approaches to enhancing bird species classification but also pose challenges in scalability and generalization.

## Materials and methods

**Fig. 1:**
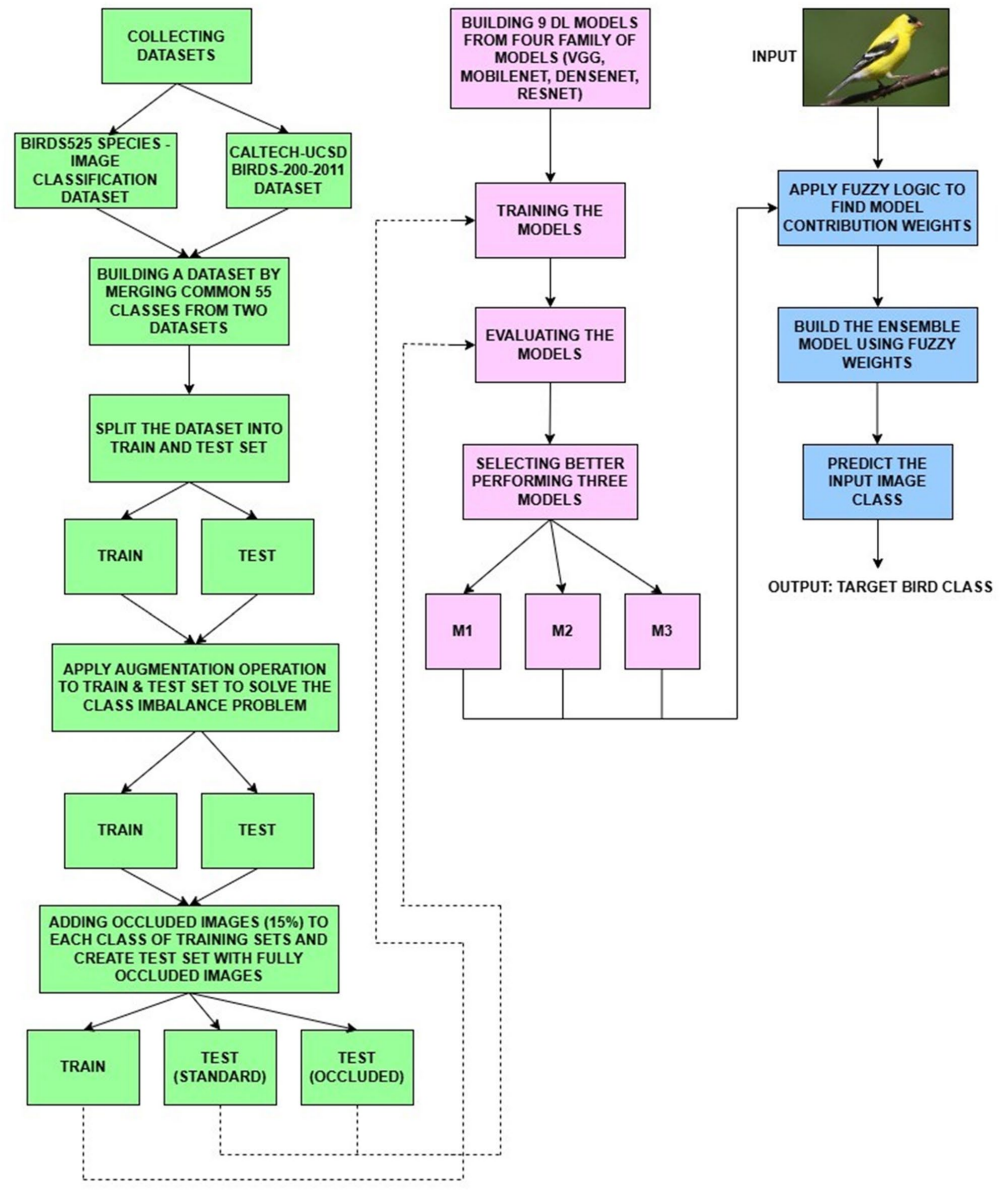
Process Pipeline to show the workflow of the proposed Bird Species Classification system.

This study aims to classify bird species using deep learning techniques trained on a dataset that includes both standard and occluded bird images. Preprocessing techniques, including labeling, augmentation, and merging, are applied to the images before training models to enhance dataset instances to avoid overfitting. Ensemble learning is utilized, which includes fuzzy ensemble learning and weighted averaging; this makes use of several models to improve the performance of the bird species classification. Lastly, we assess the system’s performance using accuracy, precision, recall, F1-Score, and Average Confidence Score. The process pipeline diagrams of the proposed approach are depicted in Fig. 1.

### Data pre-processing

Two datasets were utilized in this study, where the images were organized into directories based on bird species. The first dataset^30^, sourced from the Birds525 Species- Image Classification dataset, included 525 bird species of 84,635 training images, 2,625 test images, and 2,625 validation images. The second dataset, sourced from Kaggle, the Caltech-UCSD Birds-200-2011 dataset^31^ provided by the California Institute of Technology, contained 200 bird species, consisting of 5,994 training images and 5,794 testing images.

**Fig. 2:**
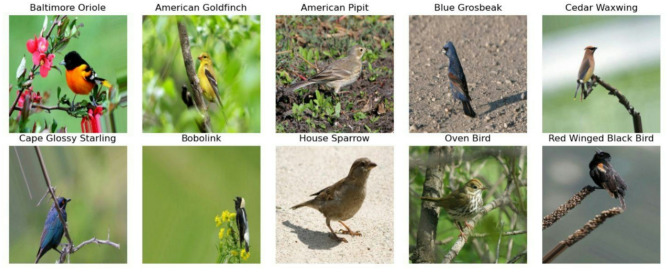
Sample bird images from different classes of the Bird Species Classification dataset.

To address the challenge of an insufficient number of instances per class for effective model training, the two datasets were merged by combining common classes into a unified dataset. In this combined dataset, 8,767 images are used from the first dataset^30^, and 2,585 images are used from the second dataset^31^, resulting in a combined dataset consisting of 11,352 images. The resulting dataset includes 55 bird species: American Goldfinch, American Pipit, American Redstart, Baltimore Oriole, Barn Swallow, Black-Throated Sparrow, Belted Kingfisher, Blue Grosbeak, Bobolink, Brown Creeper, Brewer’s Blackbird, Brown Thrasher, Cactus Wren, California Gull, Cape Glossy Starling, Cape May Warbler, Caspian Tern, Cedar Waxwing, Cerulean Warbler, Chipping Sparrow, Crested Auklet, Dark-Eyed Junco, Downy Woodpecker, Eastern Towhee, European Goldfinch, Evening Grosbeak, Gray Catbird, Gray Kingbird, Green Jay, Hooded Merganser, Horned Lark, House Sparrow, Indigo Bunting, Ivory Gull, Lazuli Bunting, Loggerhead Shrike, Mangrove Cuckoo, Northern Flicker, Northern Fulmar, Ovenbird, Painted Bunting, Pomarine Jaeger, Purple Finch, Red-Faced Cormorant, Red-Headed Woodpecker, Red-Winged Blackbird, Rose-Breasted Grosbeak, Ruby-Throated Hummingbird, Scarletanager, Spotted Catbird, Tree Swallow, Tropical Kingbird, White-Necked Raven, Yellow-Breasted Chat, and Yellow-Headed Blackbird. A few of the sample images from different classes are shown in Fig. 2.

Each class in the combined dataset accounted for about 1.5–2.5% of the total images. The dataset was divided into training and testing subsets according to the Holdout strategy, with class-wise image counts between 179 images (Mangrove Cuckoo) and 281 images (Ovenbird), with an average of about 206 images per class. In the training subset, each class accounted for about 1.5–2.5% of the dataset, whereas in the testing subset, each class accounted for about 1.8%.

**Fig. 3:**
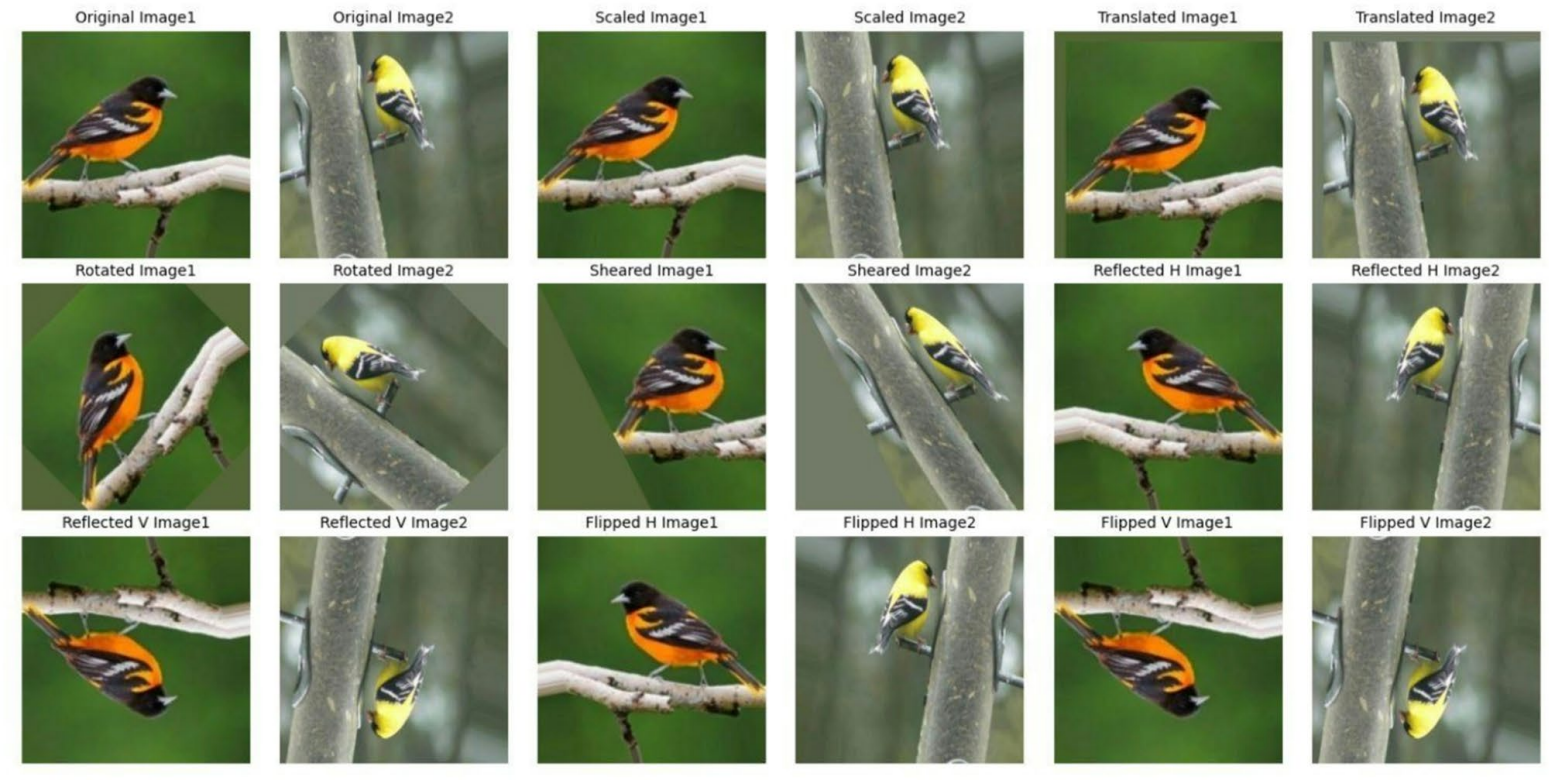
The effect of different augmentation operations on the sample images of the American Goldfinch and Baltimore Oriole bird species.

It was realized that the average number of images per class would not be enough to construct a robust model and that the variability of class distribution could influence model performance. We developed a comprehensive data augmentation approach to make the dataset more balanced and solid. Geometric transformations like scaling, translation, rotation, shearing, and photometric adjustments like horizontal and vertical reflections, and flipping are extensively applied. Using this way, the training data set is augmented to 500 images per class, and the testing data set to 100 images per class. The impact of these augmentation steps on two example classes is depicted in Fig. 3.

**Fig. 4:**
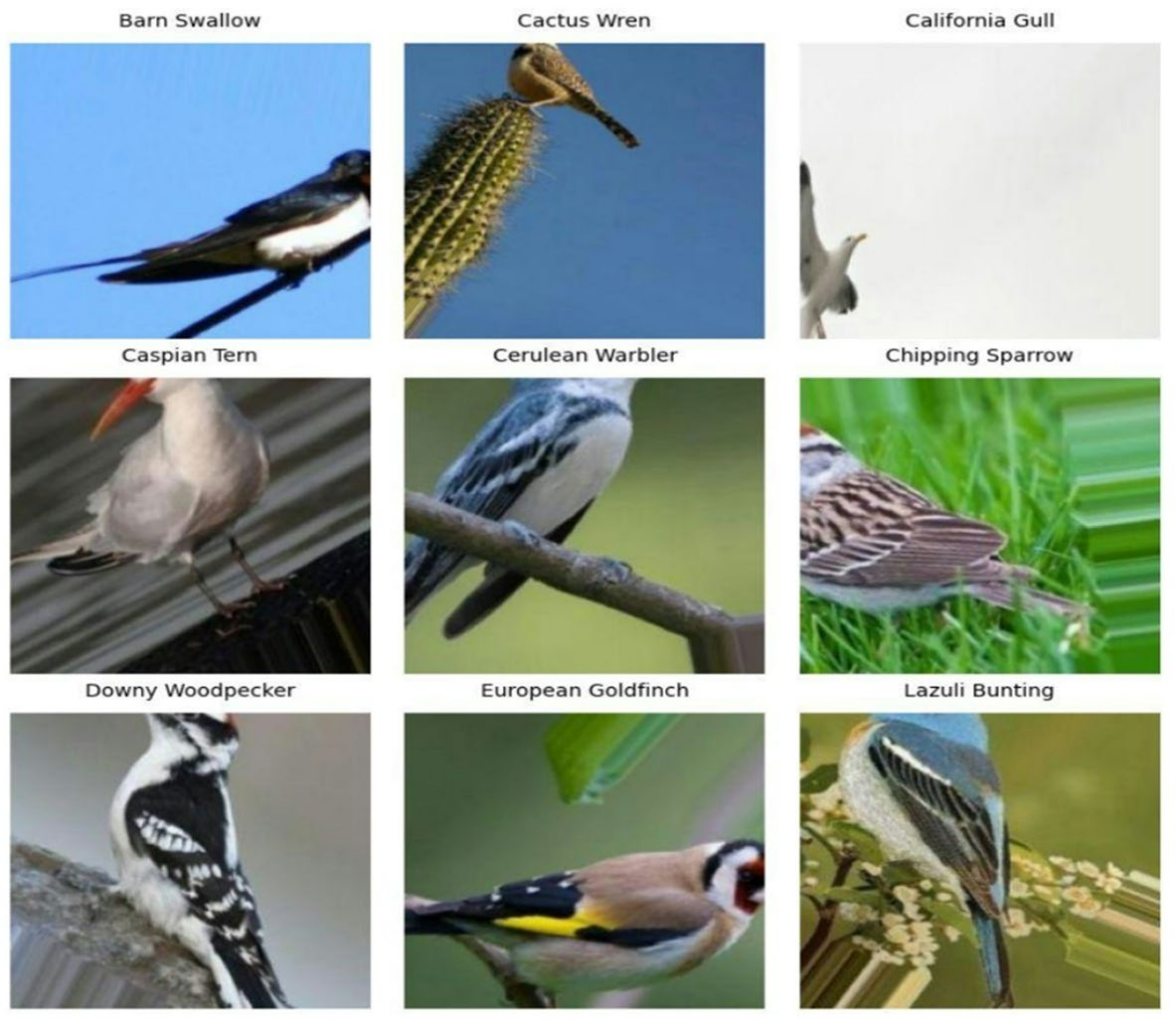
A few sample of occluded images generated using the augmentation operations.

Additionally, occluded bird object images are generated using the above-mentioned augmentation operations, enhancing the diversity of the dataset. These images of occluded birds simulate real-world conditions, such as birds partially hidden by foliage, branches, or other objects, enabling the model to recognize species accurately even when only parts of the bird are visible. A sample of the created occluded images is shown in Fig. 4. Approximately 10–15% of occluded images were incorporated into each class of the training dataset to efficiently classify images with both properly placed bird objects and occluded bird objects. Two test datasets were created: one with entirely unobstructed bird images and another exclusively containing occluded bird images. These two datasets are utilized for evaluating the efficiency of the proposed bird species classification system under various conditions.

### Building of underlying models

To build an efficient bird species classification, we have employed different variants of several families of deep learning models, such as DenseNet, ResNet, VGG, and MobileNet, since these families of deep learning models are successful in various real-time applications^32–34^. Primarily, these models are used as underlying models for extracting the feature to proceed with approaches like multi-model and ensemble learning-based systems. An extensive number of experiments is conducted on these models to extract the precise features of various classes of birds.

#### DenseNet

**Fig. 5:**
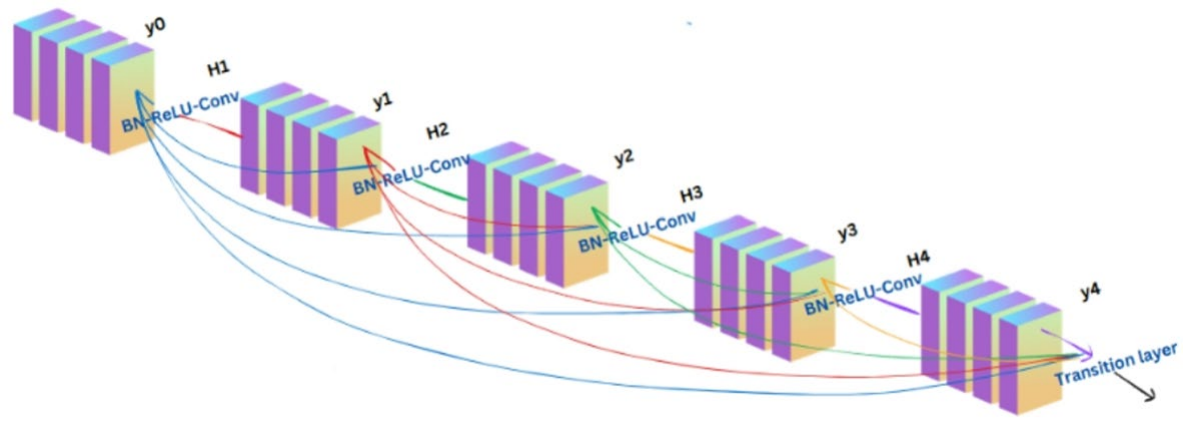
The structure of 5-layer dense block where each layer takes all preceding feature maps as input.

DenseNet, or Dense Convolutional Network, is a deep learning architecture where each layer is connected to all subsequent layers within dense blocks, promoting efficient feature reuse and gradient flow. This structure enhances learning efficiency while reducing computational redundancy and the number of parameters. Transition layers in DenseNet help to reduce computational cost and pass features efficiently by connecting dense blocks. Batch normalization, a 2 × 2 average pooling for downsampling, and a 1 × 1 convolution for channel reduction are all included in each transition layer. By compressing feature maps and limiting excessive network expansion, these layers regulate model complexity. By doing this, significant characteristics are maintained while guaranteeing a small, effective, and broadly applicable model, making DenseNet suitable for complex classification tasks^32,35^. Figure 5 illustrates the core Dense block layout schematically. This dense connection approach is represented by Eq. (1), and the transformation function used for the combined inputs at each layer is defined by Eq. 2.

1 $${y_l}={H_l}\left( {\left[ {{y_0},{y_1}, \ldots ,{y_{l - 1}}} \right]} \right)$$

2 $${H_l}\left( y \right)=Conv\left( {ReLU\left( {BN\left( y \right)} \right)} \right)$$

where ${y_l}$ represents the output layer, ${H_l}$(.) the composite function consisting of Batch Normalization (BN), Rectified Linear Unit (ReLU), conv() is the Convolution operations, and $\left[ {{y_0},{y_1}, \ldots ,{y_{l - 1}}} \right]$ represents feature maps from all previous layers 0 to l-1. This direct connection allows the network to reuse features and reduces the number of parameters compared to traditional architectures.

In this research work, we employed three DenseNet variants: DenseNet-121, DenseNet-169, and DenseNet-201. Generally, these architectures differ in their depth and computational requirements, which affect their performance and resource usage. In these variants, layers are densely connected to increase gradient flow efficiency and parameter efficiency.

DenseNet-121, characterized by 121 layers and roughly 8 million parameters, represents a compact model well-suited for smaller datasets. DenseNet-169, consisting of 169 layers, balances computational efficiency and precision. DenseNet-201, the most extensive model implemented, features 201 layers and approximately 20 million parameters, facilitating more refined feature extraction while necessitating increased computational resources^35,36^.

#### ResNet

**Fig. 6:**
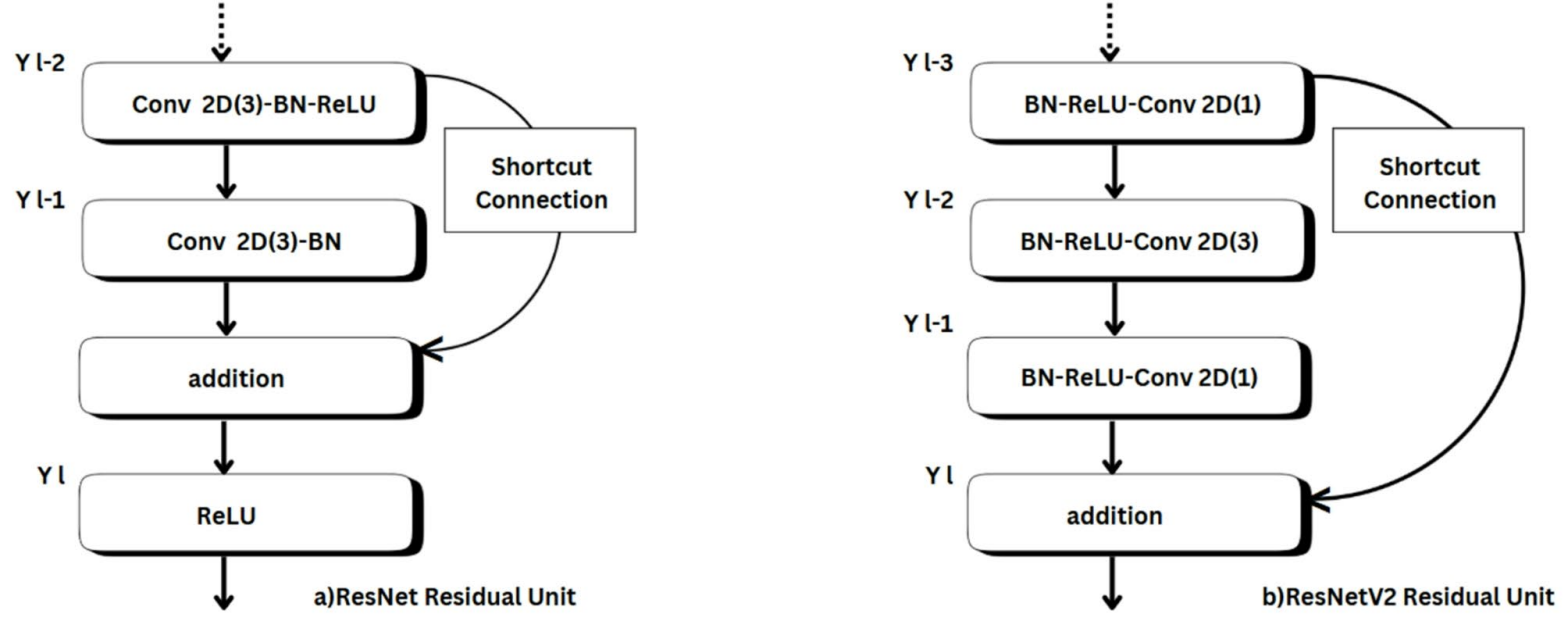
The structural diagram to depict the functionality of ResNet family models, (a) ResNet, (b) ResNetV2.

ResNet, short for Residual Network, improved deep learning through the addition of skip connections to bypass one or more layers that enable residual learning. The architecture eliminates the vanishing gradient problem and allows for improved gradient flow within deep networks. The ResNetV2 is an improvement of the original structure, where the batch normalization layer is followed by ReLU before the convolutional layers for more stable training^35,37^. Figure 6 visualizes the functionality of ResNet and ResNetV2.

The functionality differences between ResNet and ResNetV2 are represented in Eqs. (3 and 4), respectively. In ResNet (Eq. 3), the shortcut connection adds ${x_{l - 1}}$ directly to the transformed output $F\left( {{y_{l - 1}},{W_l}} \right)$ followed by applying the activation function as visible in Fig. 6. In ResNetV2 (Eq. 4), the batch normalization and ReLU are applied before the convolution operation, and the shortcut connection directly adds the input ${y_{l - 1}}$ to the output of the transformation *F*. The transformation function is now $F\left( {\sigma \left( {{y_{l - 1}}} \right),{W_l}} \right)$ with BN-ReLU-Conv ordering.

3 $${y_{1l}}=\sigma \left( {F\left( {{y_{l - 1}},{W_l}} \right)+{y_{l - 1}}} \right)$$

4 $${y_{2l}}={y_{l - 1}}+F\left( {\sigma \left( {{y_{l - 1}}} \right),{W_l}} \right)$$

where ${y_{1l}},~{y_{2l}}$ denotes the output layer of ResNet, ResNetV2, ${y_{l - 1}}$ the input to the residual block.$~{W_l}~$is the weights of the convolutional layers in this block. $F\left( {{y_{l - 1}},{W_l}} \right)~$refers to the transformation applied by a series of operations (Conv-BN-ReLU). σ (⋅) is the activation function (ReLU in ResNet).

In this research work, ResNet-50V2 and ResNet-101V2 are utilized. ResNet-50V2 has 50 layers with higher computation speed and is well-suited for mid-sized datasets. ResNet-101V2 with 101 layers has higher complex feature learning but at a higher computational load^35,37^.

#### VGG

**Fig. 7:**
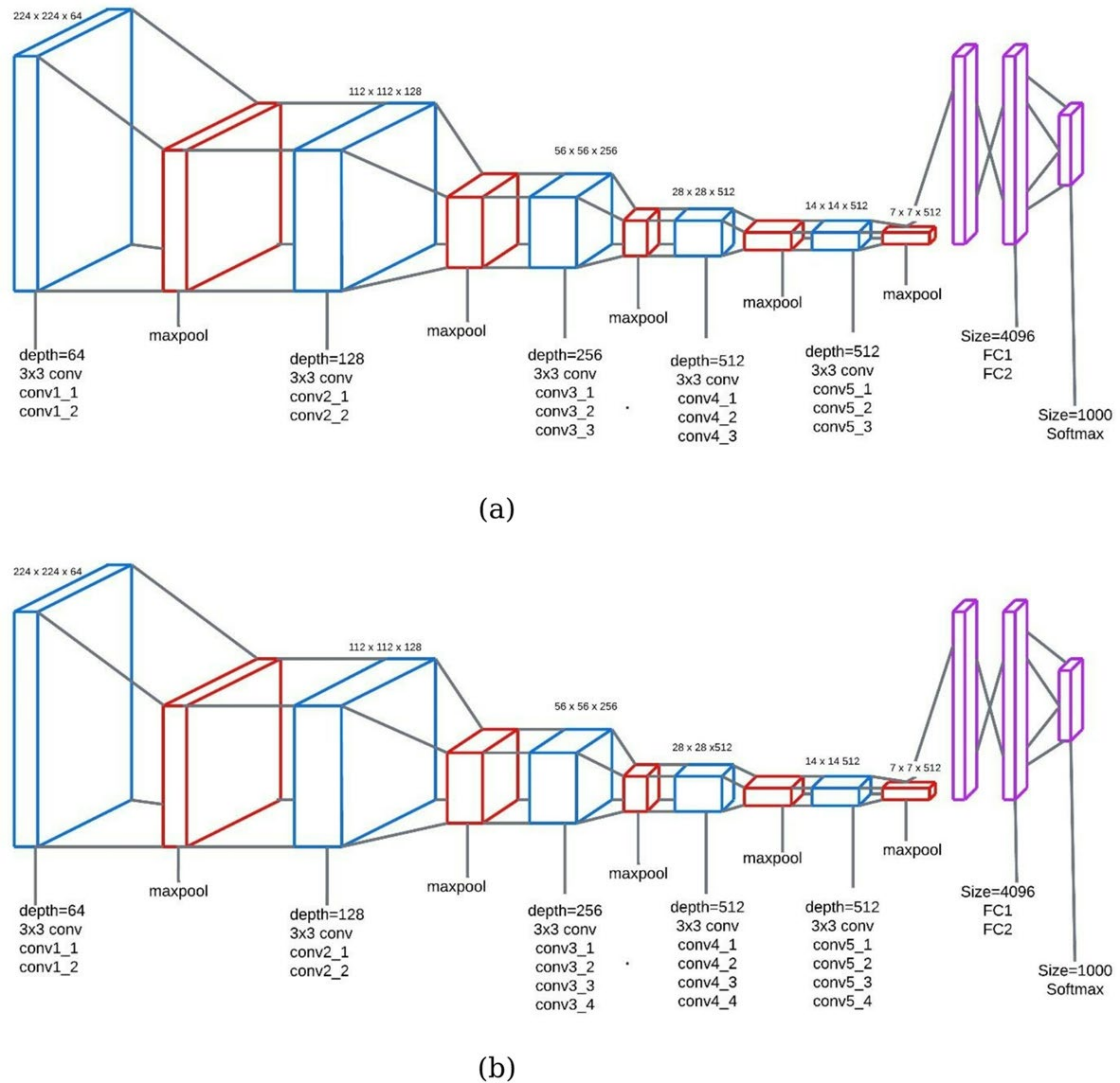
The structure of VGG family models, (a) VGG16, (b) VGG19.

VGG is one of the family of deep convolutional networks, which is relatively simple but still quite effective with a configuration of stacked 3 × 3 convolutional layers, followed by ReLU activation and max-pooling. Its design is about depth to catch hierarchical features with no shortcut connections or complex modules^35,37^. In this work, we have deployed two variants, namely VGG16 and VGG19, where VGG16 has 16 weight layers, while VGG19 adds three more convolutional layers^33,37^. The diagrammatic representation of VGG16 and VGG19 is shown in Fig. 7.

#### MobileNet

MobileNet relies on depthwise separable convolutions. It splits traditional convolutions into separate depthwise and pointwise operations, reducing parameters and the cost of computation drastically. The mathematical form of how computational complexity reduction is achieved is mentioned in Eqs. (5–10). Equations (5–7) refer to the computational cost of standard, depthwise, and pointwise (1 × 1) convolution operations, respectively. Equation (8) represents the total computational cost of depthwise-separable convolution, i.e., the sum of depthwise and pointwise cost, which is further broken down in Eq. (9), the Reduction factor mentioned in Eq. (10) measures the computational savings while using depthwise separable convolution compared to the standard version.

5 $$Cos{t_{standard}}={D_f} \times {D_f} \times {C_{in}} \times {C_{out}} \times {D_O} \times {D_O}$$

6 $$Cos{t_{depthwise}}={D_f} \times {D_f} \times {C_{in}} \times {D_O} \times {D_O}$$

7 $$Cos{t_{pointwise}}=1 \times 1 \times {C_{in}} \times {C_{out}} \times {D_O} \times {D_O}$$

8 $$Cos{t_{seperable}}=Cos{t_{depthwise}}+Cos{t_{pointwise}}$$

9 $$Cos{t_{separable}}=D_{f}^{2} \times {C_{in}} \times D_{o}^{2}+{C_{in}} \times {C_{out}} \times D_{o}^{2}$$

10 $$Reduction=\frac{{Cos{t_{separable}}}}{{Cos{t_{standard}}}}=\frac{{D_{f}^{2} \times {C_{in}} \times {C_{out}}}}{{D_{f}^{2} \times {C_{in}}+{C_{in}} \times {C_{out}}}}$$

where $Df$ is the filter size for the convolutional kernel, ${C_{in}}$ represents the number of input channels, ${C_{out}}$ represents the number of output channels, $D_{o}^{i}$ represents the output feature map size. $Cos{t_{standard}}$ refers to the computational cost of a standard convolutional layer, whereas $Cos{t_{depthwise}}$ refers to the cost of depthwise convolution, where each filter is applied to a single input channel. $Cos{t_{pointwise}}$ denotes the cost of a pointwise (1 × 1) convolution used to combine the outputs of depthwise convolution. $Cos{t_{separable}}$ is the total cost of the depthwise separable convolution, calculated as the sum of depthwise and pointwise costs.

The MobileNet variants, namely MobileNetV2 and MobileNetV3_small, are implemented in this study. MobileNetV2 introduces inverted residual blocks where the skip connections connect a narrower layer, while an expansion layer uses a larger convolution filter. The MobileNetV2 architecture, coupled with the inclusion of a linear bottleneck, enhances the efficiency of the operations and the representational capacity. MobileNetV3_small elaborates on these details with squeeze-and-excitation modules modifying channel-wise feature maps adaptively, combining with neural architecture search that optimizes the trade-off between performance and complexity^35,36^.

**Fig. 8:**
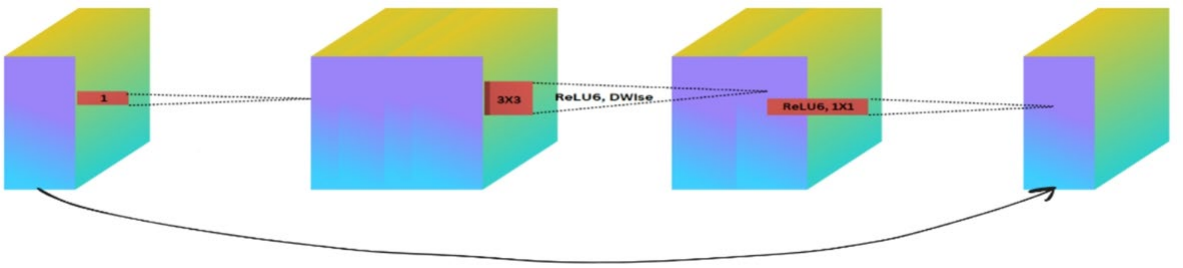
The structure of the MobileNetV2 model block.

**Fig. 9:**
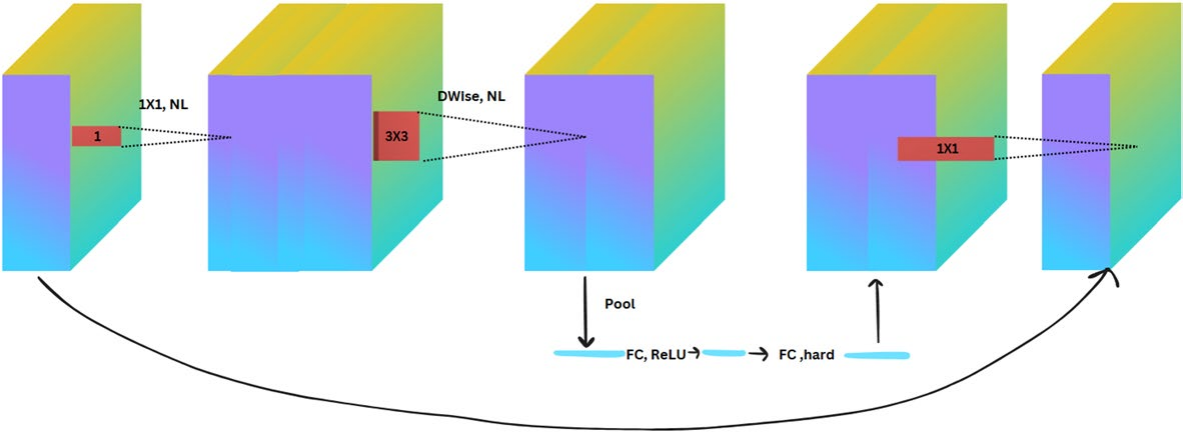
The structure of the MobileNetV3_small model block.

Mathematically, we define the differences between MobileNetV2 and MobileNetV3 small in the following Eqs. (11–14). MobileNetV2 uses inverted residual blocks with linear bottlenecks, as seen in Fig. 8. The struture of MobileNetV3_small is visualized in Fig. 9.

11 $$y=x+F\left( {x,W} \right)$$

12 $$h - swish\left( x \right)=x \cdot \frac{{ReLU6\left( {x+3} \right)}}{6}$$

13 $${\text{Squeeze}}:{z_c}=\frac{1}{{H \times W}}\mathop \sum \limits_{{i=1}}^{H} \mathop \sum \limits_{{j=1}}^{W} {x_{c,i,j}}$$

14 $$Excitation:scale\left( {{x_c}} \right)=\sigma \left( {{W_2}\delta \left( {{W_1}{z_c}} \right)} \right) \cdot {x_c}$$

Equation (11), illustrates inverted residual expanding input channels with a 1 × 1 convolution, performing depthwise convolution (3 × 3), and then projecting back to a lower-dimensional space with another 1 × 1 convolution. The Activation Function uses ReLU6 for non-linearity in both depthwise and pointwise convolutions. Here, *x* is the input, *y* is the output of the residual block, $F\left( {x,W} \right)$ is a depthwise separable convolution operation with ReLU6 activation applied.

Equation (12) defines the Hard-Swish activation function, where ReLU6 is a clipped ReLU function. In Eq. (13), the squeeze operation computes a global average pooling$~~{z_c}~$over the spatial dimensions H and W of each channel. Equation 14 describes the excitation operation, where the output ${z_c}$ is passed through two fully connected layers with weights W1 and W2, ReLU activation δ(⋅), and sigmoid function σ(⋅), producing a scale that modulates the channel-wise feature ${x_c}$.

### Bird classification using multimodal approaches

Multimodal methodologies combine multiple feature maps generated by various underlying deep learning models to enhance predictive precision and include more comprehensive contextual information. This multimodal approach is conducted in three methods, namely, the Hybrid approach, simple Ensemble learning approach, and Fuzzy-based Ensemble learning approach^38^.

#### Hybrid approach

Random Forest reduces overfitting and effectively handles high-dimensional data by using ensemble learning to give robustness. For limited and complex datasets, Support Vector Machine (SVM) works well because it guarantees optimal decision bounds. Adaptability to changes in data distribution is enhanced by KNN’s (K-Nearest Neighbours) ability to record local data structures as a distance-based classifier. The hybrid technique improves robustness, accuracy, and adaptability by combining these algorithms with CNN models, which makes it ideal for identifying a variety of bird species in a range of environmental circumstances.

In the Hybrid Approach, the features of the training dataset are extracted using the top three better-performing trained models. These extracted features are concatenated to produce a feature pyramid and utilized to train the Machine Learning Algorithms-based classifiers like Random Forest, SVM, and KNN. After the classifiers are trained, their performance is evaluated using the extracted dataset features.

**Fig. 10:**
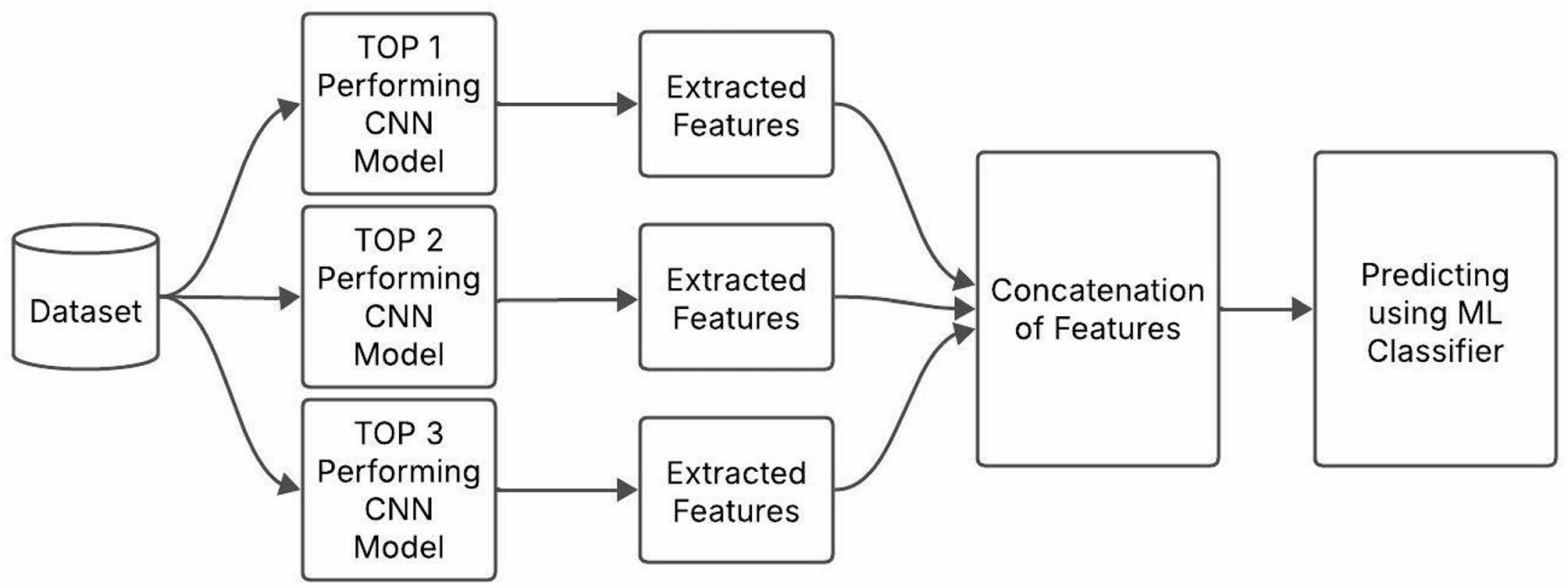
The structure of the hybrid approach used in this research work.

This method combines the diversity in feature extraction by the top three better-performing models and the classification strength of machine-learning-based classifiers to attain the precise classification of bird species. The functionality of the hybrid approach is observed in Fig. 10 and Algorithm 1.

**Figure:**
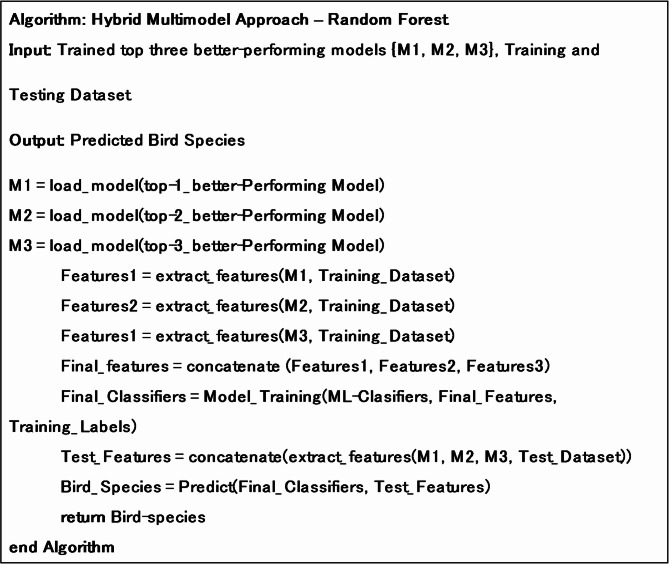
**Algorithm 1**. Functionality of the hybrid approach using Random Forest.

**Figure:**
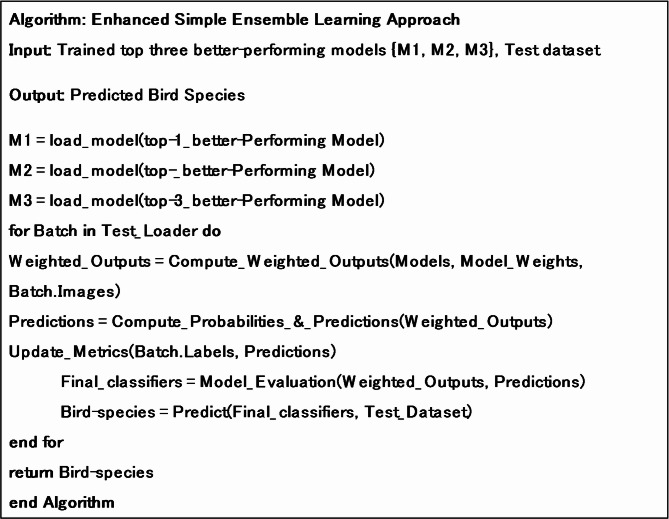
**Algorithm 2**. Functionality of the simple ensemble learning approach.

#### Ensemble approach

The ensemble approach is conducted using a heterogeneous group of models, where the top three better-performing models are included. In this ensemble approach, the approach integrates predictions coming from multiple architectures of models for the improvement of classification accuracy and generalizability.

The ensemble method combines the strengths of these models by combining their outputs through majority voting or weighted averaging, thus improving overall performance on precise bird species classification. Since it incorporates the top three better-performing models across the various families of models, it is crucial to capture subtle differences across the different bird species^39^. The functionality of the simple ensemble learning approach is observed in Algorithm 2.

#### Fuzzy-based ensemble learning approach

**Fig. 11:**
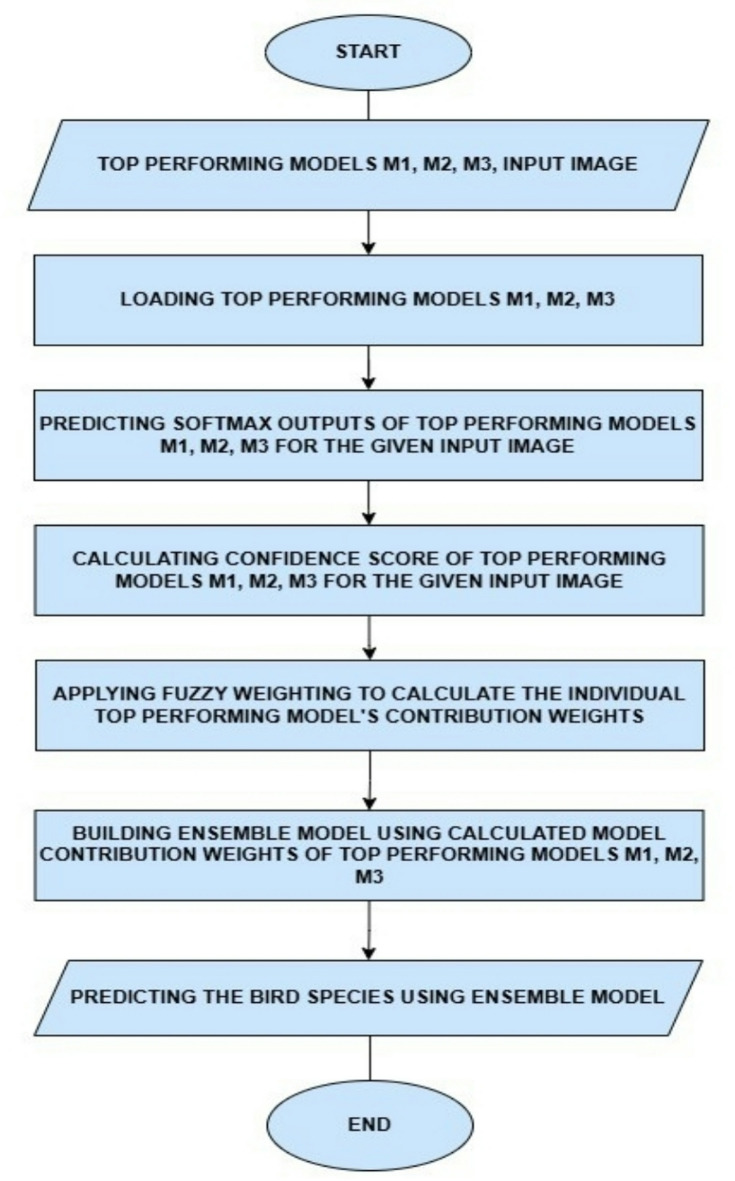
Flow chart of the Fuzzy-based Ensemble Learning Approach proposed in this work.

In the Fuzzy-based Ensemble Learning approach, predictions from multiple models are combined using fuzzy logic to assign dynamic weights based on the confidence levels of each model. Unlike simple ensemble learning, which assigns static or pre-determined weights, the fuzzy-based approach dynamically adjusts weights, allowing it to better handle uncertainty and variability in model performance^40^. At evaluation time, SoftMax probabilities are computed for each model. The average confidence scores are used as inputs for the fuzzy inference system to calculate dynamic weights. All these weights adaptively balance the contribution of the models while ensuring that the more reliable models contribute to final decisions. Weighted probabilities aggregate towards predicting final bird species, enabling precise classification with improved overall performance. The functionality of this proposed fuzzy-based approach is mentioned in Algorithms 3 and 4, and the corresponding flowchart to describe the functionality is represented in Fig. 11.

Mathematically, we can define the ensemble learning and fuzzy weighting approach from the Eqs. (15–18). This method enhances the robustness of final predictions by dynamically modifying model contributions using fuzzy logic according to each model’s confidence.

15 $${p_i}\left( x \right)=SoftMax\left( {Mi\left( x \right)} \right)=\left[ {p_{i}^{{\left( 1 \right)}}\left( x \right),p_{i}^{{\left( 2 \right)}}\left( x \right), \ldots ,p_{i}^{{\left( K \right)}}\left( x \right)} \right]~$$

16 $${c_i}\left( x \right)=max{_k}\left( {p_{i}^{{\left( K \right)}}\left( x \right)} \right)$$

17 $${w_i}\left( x \right)=FuzzyInference\left( {{c_1}\left( x \right),{c_2}\left( x \right),{c_3}\left( x \right)} \right)$$

18 $$EP\left( x \right)=\mathop \sum \limits_{{i=1}}^{3} {w_i}\left( x \right) \cdot {p_i}\left( x \right)$$

where M_i_, i ∈ {1,2,3}, denotes the model, K the number of classes, x - input image. The vector ${p_i}\left( x \right)$ defines the model’s belief in each class label. The confidence score of each model M_i_ is computed in Eq. (16). as the maximum SoftMax output among all the classes. This scalar value ${c_i}\left( x \right)$ represents how confident model M_i_ is in its top prediction for input x. Equation (17) derives the dynamic weights ${w_i}\left( x \right)$ using fuzzy logic rules, individual model confidence scores ${c_i}\left( x \right).$ Eq. (18) defines the final ensemble probability vector EP(x) as the weighted sum of the SoftMax outputs ${p_i}\left( x \right)$ of the individual top-performing models M_i_​, where the weights ${w_i}\left( x \right)$ are dynamically computed using a fuzzy weighting mechanism in Eq. (18).

**Figure:**
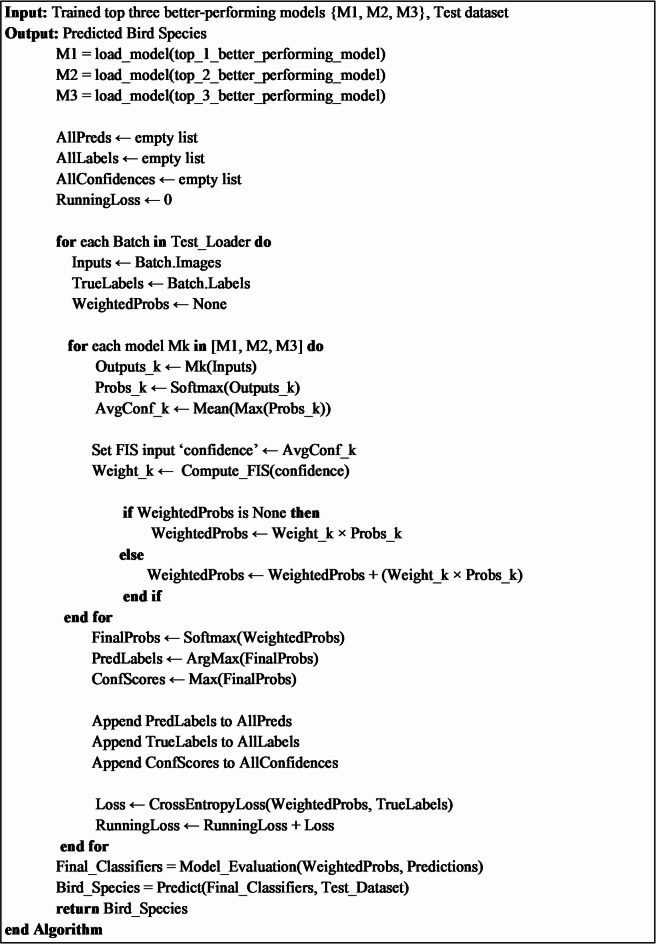
**Algorithm 3**. Functionality of Fuzzy-based Ensemble Learning.

**Figure:**
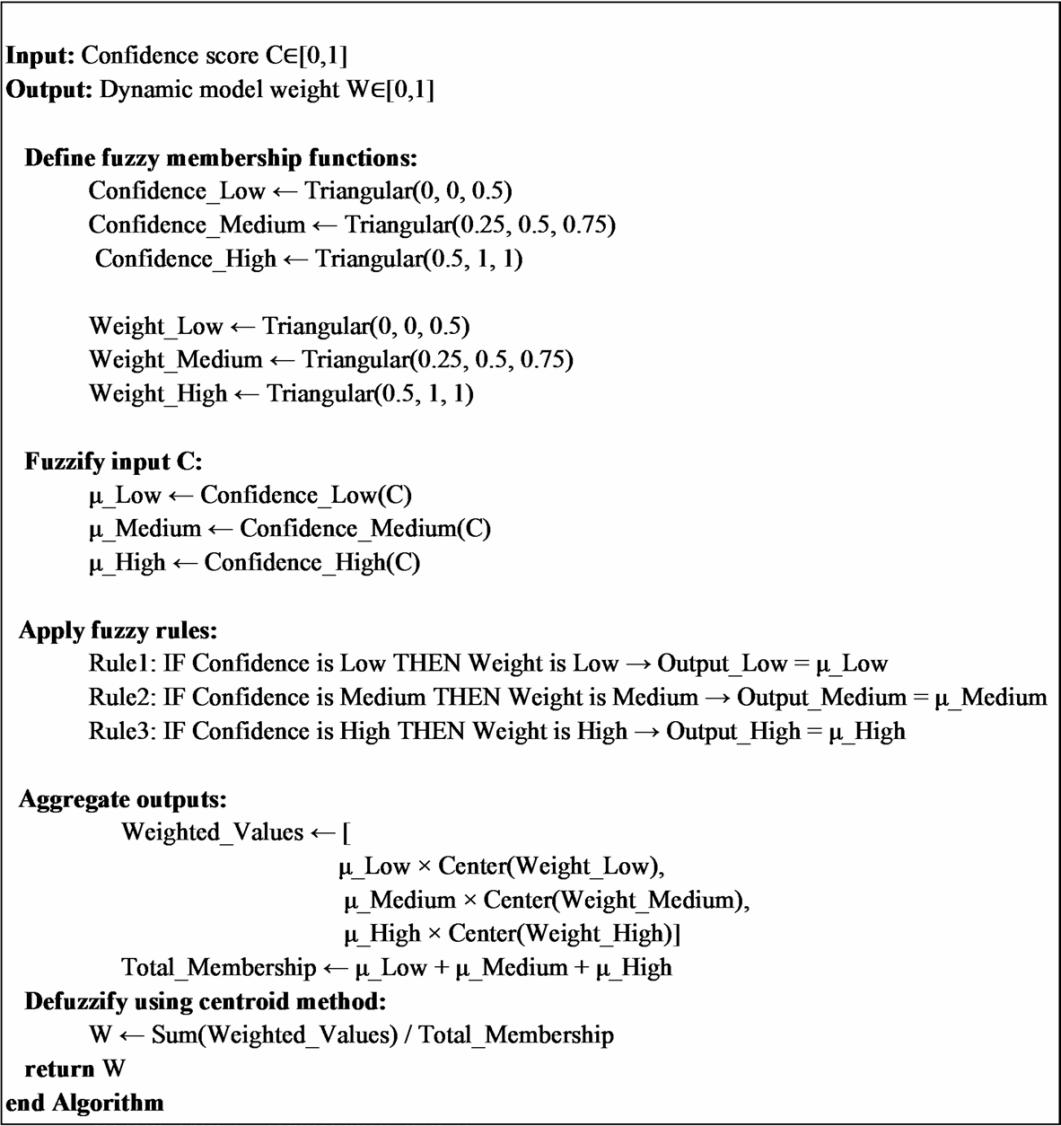
**Algorithm 4**. Compute_FIS(confidence).

## Experimental environment and evaluation metrics

An overview of the experimental environment and the evaluation metrics that were used to evaluate the performance of the proposed system is provided below:

### Experimental environment used for developing the underlying deep learning models

The proposed architecture is implemented using the PyTorch framework. The experiments are conducted on the Google Colab platform, which provides an NVIDIA Tesla T4 GPU, 16 GB of RAM, and 77 GB of disk space. All the proposed deep learning architecture variants are trained for 50 epochs, with a batch size of 16 images per batch. Consequently, each epoch comprises 860 training steps and 163 validation steps.

For this multi-class classification task involving 55 bird species, the categorical cross-entropy loss function is utilized. This function is ideal for multi-class problems as it calculates the cross-entropy between the true target labels and the predicted values, effectively guiding the model toward accurate predictions. To minimize the loss, the RMS Prop optimizer is employed. Unlike the typical stochastic gradient descent (SGD) optimizer, RMSprop learns the learning rate automatically through a moving average of the squared gradients to avoid catastrophic updates and convergence. It is best utilized in non-stationary targets and functions optimally in mini-batch training settings.

In this experiment, the RMSprop optimizer with a fixed learning rate of 1e-5 is applied for all 50 epochs. The learning rate is a helpful parameter in deep learning, where it controls how rapidly the model updates its parameters with each training batch.

In this environment, features are extracted using CNN and aggregated with RF, KNN, and SVM to leverage diverse learning paradigms for robust classification and better predictive capacity. Ensemble learning uses a weighted averaging technique to fuse predictions from distinct models to attain maximum overall accuracy and reliability. Fuzzy-based ensemble learning is also used to counteract ambiguity and uncertainty during prediction to aid decision-making using adaptive model aggregation.

### Evaluation metrics

There are five evaluation metrics used to evaluate the model’s efficiency for bird species classification.

- Accuracy (A).
- Precision (P).
- Recall (R).
- F1 Score (FS).
- Average Confidence Score (ACS).

Accuracy (A) is the ratio of the correctly classified images to the total number of images, which indicates the overall efficiency of the model in making the right predictions. The precision (P) is given by the ratio of correctly classified images to the images classified as positive. The recall (R) is given by the rate of actual positive predictions made correctly. The F1-score (FS) is a method of calculating precision and recall together, and it is given as the harmonic mean of precision and recall. The average confidence score (ACS) is the mean probability that the model assigns to its output class, and it indicates the confidence of its predictions. The mathematical form of the above metrics is mentioned in Eqs. (19–23).

19 $$A= \frac{{TP+TN}}{{TP+TN+FP+FN}}$$

20 $$P = \frac{{TP}}{{TP+FP}}$$

21 $$R = \frac{{TP}}{{TP+FN}}$$

22 $${\text{FS }} = {\text{ 2}}~ \times \frac{{P~X~R}}{{P + R}}$$

23 $${\text{ACS~}}={\text{~}}\frac{1}{N}{\text{~}}\mathop \sum \limits_{{i=1}}^{N} ci$$.

where, A represent Accuracy, P represent Precision, R represent Recall, FS represent F1 Score, ACS represent Average Confidence Score, TP represent True Positive, TN represent True Negative, FP represent False Positive, FN represent False Negative, N represent Total Number of Predictions, ci represent confidence score assigned to the predicted class for the i-th sample.

## Results and discussion

The analysis of results attained in this bird species classification work is categorized and subdivided as follows,

- Performance Comparisons of Stand-alone Models with Standard Bird Objects.
- Performance Comparisons of Stand-alone Models with Occluded Bird Objects.
- Performance Comparisons on the Multi-model Approach.
- Performance Comparisons on the existing state-of-the-art research.
- Ablation Study.

### Performance comparisons of stand-alone models with standard bird objects

**Fig. 12:**
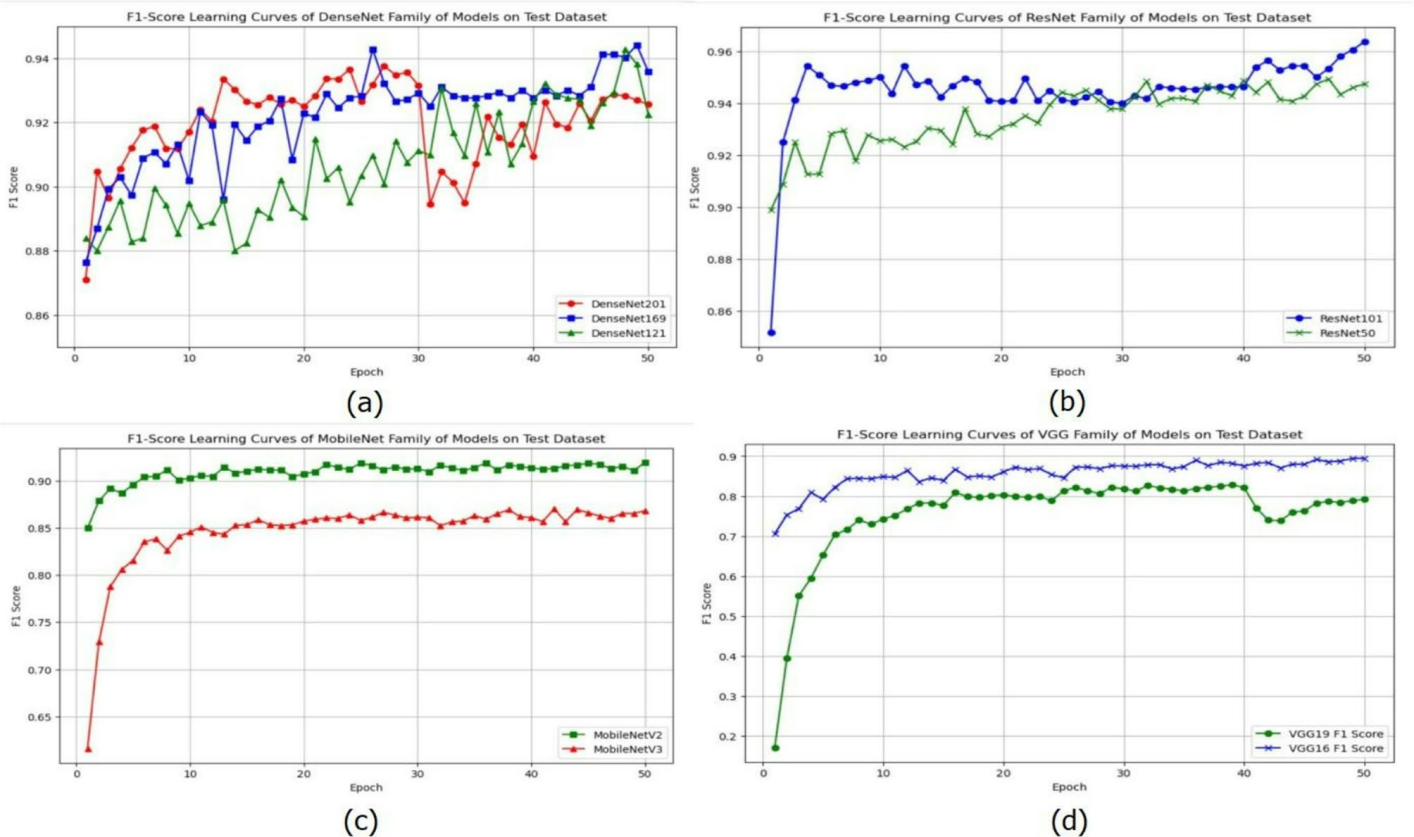
F1-score learning curves of the different family models employed in this research work, (a) DenseNet family of models, (b) ResNet family of Models, (c) MobileNet Family of models, (d) VGG family of models.

In this research, we employed four families of CNN models- DenseNet, ResNet, VGG, and MobileNet for building a model for the bird species classification system. From these four families of models, nine variants of models, such as DenseNet121, DenseNet169, DenseNet201, ResNet50v2, ResNet101v2, VGG16, VGG19, MobileNetV3Small, and MobileNetV2, are employed. All these models are loaded with ImageNet pre-trained weights to incorporate the transfer learning. Later, these models are fine-tuned using the training dataset mentioned in the experimental environment mentioned in section “Experimental environment used for developing the underlying deep learning models”. Here, the transfer learning is incorporated with *trainable = true* configuration, where all the layer weights of the models are fine-tuned. The metric scores obtained by all the models on the training and testing datasets (standard images) are presented in Table 2. The learning curves of validation time F1-Score for all the family models are visualized in Fig. 12. Analyzing the F1-score over epochs is important since it ensures your model’s true learning capability instead of exploiting dataset biases.

Based on the observations from Table 2 comparisons and learning curves from Fig. 12, the models DenseNet121, DenseNet169, and ResNet101v2 have been achieving better scores across all the metrics in the test dataset with standard bird object due to their architectural benefits. ResNets use residual connections, which enable training networks more deeply and alleviate vanishing gradient issues, whereas DenseNet uses concatenation connections that have enhanced feature reuse and strong gradients throughout the network. Specifically, DenseNet121 achieved an accuracy of 95.07%, an F1 score of 95.13%, and an average confidence score of 97.78%; DenseNet169 achieved an accuracy of 94.96%, an F1 score of 94.94%, and an average confidence score of 98.31%; and ResNet101v2 achieved an accuracy of 96.04%, an F1 score of 96.02%, and an average confidence score of 97.78%, on test dataset demonstrating their robustness and reliability in the bird species classification task.

**Table 2:** The performance comparison of the underlying standalone CNN models on standard bird species images.

S.No	Family	Model name	Training metrics				Testing Dataset with Standard Bird Objects				
			A (%)	*P* (%)	*R* (%)	FS (%)	A (%)	*P* (%)	*R* (%)	FS (%)	ACS (%)
1	DenseNet	DenseNet121	99.99	99.99	99.99	99.99	95.07	95.21	95.06	95.13	97.78
		DenseNet169	99.99	99.99	99.99	99.99	94.96	95.04	94.85	94.94	98.31
		DenseNet201	99.91	99.91	99.91	99.91	93.93	94.28	93.93	94.1	96.76
2	ResNet	ResNet50V2	99.98	94.76	99.98	97.3	94.77	94.83	94.71	94.77	98.22
		ResNet101V2	99.99	99.99	99.99	99.99	96.04	96.09	95.95	96.02	97.78
3	VGG	VGG16	99.97	99.96	99.96	99.96	90.07	90.44	90.1	90.22	97.97
		VGG19	99.78	99.78	99.78	99.78	87.27	88.03	87.27	87.65	96.54
4	MobileNet	MobileNetV3-small	97.89	97.91	97.9	97.9	86.54	54.69	87.11	67.19	95.42
		MobileNetV2	99.89	99.9	99.9	99.9	91.71	92.22	91.71	91.96	96.72

A: Accuracy, P: Precision, R: Recall, FS: F1-Score, ACS: Average Confidence Score.

**Fig. 13:**
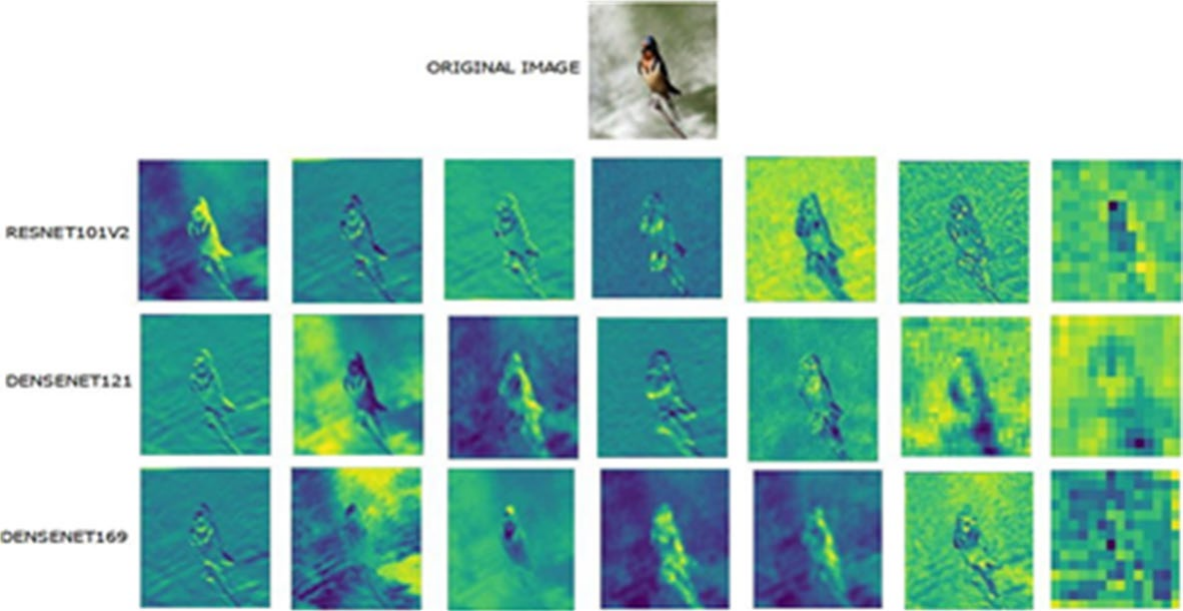
Feature-map visualization by the top three better-performing models (DenseNet121, DenseNet169, and ResNet101v2) for a sample image from the Barn Swallow Bird Species class.

**Fig. 14:**
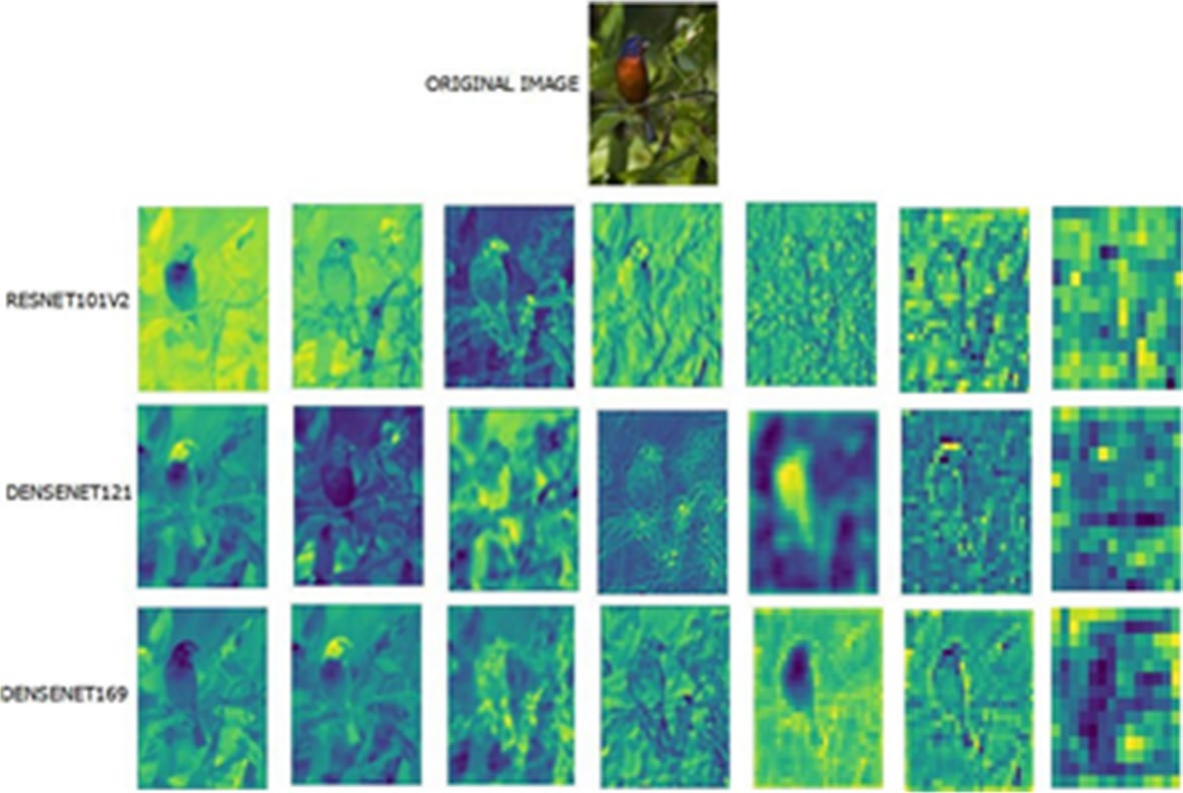
Feature-map visualization by the top three better-performing models (DenseNet121, DenseNet169, and ResNet101v2) for a sample image from the Painted Bunting Bird Species class.

To strengthen these results, feature mapping has been carried out to visualize and track the features learned at various layers of the best-performing models. Random feature maps from the convolutional layers, organized from earlier to deeper layers, are plotted to provide insights into the kinds of patterns learned and their contribution to the decision-making process. This makes the models more interpretable, and it becomes easier to find out which specific features from the images are responsible for the bird species classification. Feature maps generated by the top three better-performing models (DenseNet121, DenseNet169, and ResNet101v2) for a sample image from the two-class bird species, namely Barn Swallow and Painted Bunting, are visualized in Figs. 13 and 14.

### Performance comparisons of stand-alone models with occluded bird objects

In their native habitat, birds are frequently partially obscured by leaves, branches, or other environmental features. These real-world circumstances are simulated by different augmentation operations to evaluate the model on obscured images, which guarantees that the system can function dependably outside of controlled settings. Additionally, the occlusion trials show how successfully the model employs distinguishing characteristics like wing patterns, beak form, or plumage colour rather than depending on unimportant features of the bird objects.

Thus, experiments were conducted to test the robustness of the model’s performance on occluded bird object and their ability to handle incomplete or occluded inputs, which is an important factor in real-world applications. To implement this, we have created a separate test dataset of 400 images of 55 classes using various augmentation operations, which contains occluded bird images of each bird species class. Later, all these trained models are evaluated using this Test Dataset-2 with occluded bird objects, and the corresponding results are recorded in Table 3 for performance comparison.

**Table 3:** The performance comparison of underlying standalone CNN models on occluded bird species images.

S.No.	Family	Model name	Testing dataset with occulded bird objects				
			A (%)	*P* (%)	*R* (%)	FS (%)	ACS (%)
1	DenseNet	DenseNet121	91.77	90.69	91.65	91.17	93.45
		DenseNet169	92.14	88.79	91.57	90.16	97.72
		DenseNet201	88.61	88.92	91.46	90.17	93.64
2	ResNet	ResNet50V2	89.81	87.07	91.71	89.33	94.89
		ResNet101V2	91.41	91.75	91.44	91.59	95.46
3	VGG	VGG16	77.22	79.39	81.84	80.6	90.19
		VGG19	75.64	74.87	74.21	74.54	89.56
4	MobileNet	MobileNetV3-small	74.36	74.32	74.21	74.26	89.12
		MobileNetV2	84.18	80.6	84.22	82.37	94.78

A: Accuracy, P: Precision, R: Recall, FS: F1-Score, ACS: Average Confidence Score.

According to the observations from Tables 2 and 3, a decline in performance is evident across all models when tested on the occluded dataset. Even the better-performing models derived from Table 2—DenseNet121, DenseNet169, and ResNet101v2— also show this pattern. The following deviations are observed when comparing their performance with Table 2: accuracy decreases by 3.3%, F1-score by 3.96%, and average confidence score (ACS) by 4.33% for DenseNet121; accuracy, F1-score, and ACS for DenseNet169 are 1.92%, 4.78%, and 0.59%, respectively; and accuracy decreases by 4.63%, F1-score by 4.43%, and ACS by 2.32% for ResNet101v2. These findings highlight that standalone models struggle with occluded images, which is a significant challenge in real-world scenarios where such images are common. A multimodal approach is utilized to address this issue by using the advantages of models that are performing better on the test dataset with Standard images (Table 2) to increase robustness and boost classification performance in the presence of occlusion.

### Performance comparisons on the multi-model approach

Usually, a standalone CNN may inadvertently develop biases toward specific features or patterns in the training dataset^41^. A multi-model approach reduces such biases since different models have different biases and strengths. While aggregating their predictions or feature maps, the robustness, generalization, and performance of the system will be improved. Also, the multi-CNN model approach can specialize in learning different features or aspects from the images. We have employed three multi-model approaches in this research work, namely.

A. Hybrid Approach,
B. Simple Ensemble Learning Approach,
C. Fuzzy-based Ensemble Learning Approach.

These approaches were designed to leverage the strengths of the better-performing models, DenseNet121, DenseNet169, and ResNet101v2, to enhance the precision and robustness of bird species classification, especially for occluded bird objects. At first, the Hybrid Approach concatenates the extracted features of these top three better-performing models and combines these concatenated features with a secondary ML-based classifier, namely Random Forest, Support Vector Machine (SVM), and K-nearest neighbor (KNN), to enhance the classification by aggregating diverse feature representations. Table 4 records the results of the performance comparison of the Hybrid Approach model with different classifiers, namely Random Forest, Support Vector Machine, and K Nearest Neighbour on the Standard images and Occluded bird images dataset.

**Table 4:** The performance comparison of different ML-based classifier combinations used in the with the top three better-performing models with hybrid approach on standard images and occluded images.

Classifier	Training dataset				Testing dataset with standard bird objects					Testing dataset with occluded bird objects				
	A (%)	*P* (%)	*R* (%)	FS (%)	A (%)	*P* (%)	*R* (%)	FS (%)	ACS (%)	A (%)	*P* (%)	*R* (%)	FS (%)	ACS (%)
Random Forest	99.99	99.99	99.99	99.99	95.61	95.88	95.61	95.74	96.4	93.41	93.43	93.41	93.42	95.21
SVM	99.99	99.99	99.99	99.99	96.5	96.66	96.5	96.58	97.78	92.14	93.95	92.14	93.04	93.24
KNN	99.99	99.99	99.99	99.99	96.65	96.79	96.65	96.72	99.16	92.77	93.9	92.77	93.33	94.36

A: Accuracy, P: Precision, R: Recall, FS: F1-Score, ACS: Average Confidence Score.

According to the observations from Table 4, applying the Hybrid approach by combining the better-performing models (DenseNet121, DenseNet169, and ResNet101v2- derived from Table 2) has resulted in an improvement in classification performance when compared to standalone models. The Hybrid approach’s performance was assessed on both standard and occluded bird object images. Compared to standard images, the hybrid approach results in a notable improvement in classification performance on Occluded images. We find that the Hybrid Approach is robust in handling partial occlusions, as evidenced by the improvement in accuracy, F1-score, and confidence score for occluded images as compared to standalone models (Table 3).

In particular, Random Forest outperforms SVM and KNN in terms of accuracy (93.41%), F1-score (93.42%), and confidence score (95.21%) for occluded images. According to these results, combining the extracted features of the top three better-performing models with machine-learning classifiers improves classification accuracy and increases the method’s resistance to difficult real-world situations where images are frequently obscured. However, despite these gains, the hybrid approach did not achieve optimal performance on occluded images, thus, we use an ensemble learning approach to improve robustness and accuracy.

In the other two multi-modal approaches, we have also employed two ensemble-based learning approaches, namely Simple Ensemble Learning and Fuzzy-based Ensemble Learning. The straightforward ensemble learning strategy assigns fixed weights statistically to each model depending on their performance, to be adaptable and achieve higher overall accuracy. Meanwhile, the Fuzzy-based Ensemble Learning Strategy applies fuzzy logic in assigning adaptive weights to the models, making them more adaptable and accurate for bird species classification. Generally, these methods indicate the capability of multi-CNN methods in resolving complicated image-classification problems. Table 5 discusses the performance of simple ensemble learning and fuzzy-based ensemble learning on the standard bird object image and the occluded bird object images test dataset.

The Hybrid Approach’s moderate generalization may still be subject to feature extractor biases, which might limit its capacity to adjust dynamically to variations in the data. Ensemble Learning, on the other hand, efficiently raises overall classification accuracy by utilizing several model predictions to provide excellent generalization. The performance attained by the different ensemble learning methods on the Standard Bird Objects and Occluded Bird Objects test dataset is recorded in Table 5.

The performance of the proposed fuzzy-based and straightforward ensemble approaches was assessed using the powerful comparison baselines provided by the Random Forest and XG-Boost ensemble learning algorithms. The efficiency of these techniques in addressing complicated, high-dimensional classification issues is well known. By merging the predictions of several decision trees, Random Forest successfully reduces overfitting and increases model stability and generalization. Known for its accuracy and scalability, XG-Boost uses gradient boosting and regularization to improve accuracy and robustness, especially when dealing with noisy or unbalanced data. Because of their inclusion, a thorough and insightful evaluation of the suggested ensemble techniques’ performance under both typical and difficult circumstances is made possible.

According to Table 5, the performance of the proposed Fuzzy-Based Ensemble Learning method shows a remarkable improvement compared to conventional ensemble methods, such as Simple Ensemble Learning, Random Forest Ensemble, and XGBoost Ensemble Learning, on both normal and occluded bird object testing datasets. Particularly, the fuzzy-based model obtained the highest accuracy, which improved by 1.17% concerning the Simple Ensemble Learning method under normal conditions (98.73% vs. 97.56%) and by 1.01% under occluded conditions (95.78% vs. 94.77%). When precision, recall, and F1-score are concerned, the fuzzy-based method performed better than the rest by significant margins. For example, its F1-score was 2.17% better in normal situations (98.75% compared to 96.05% for Random Forest) and 6.4% better in occluded situations (95.10% compared to 88.70% for Random Forest), indicating that it can deal with difficult situations involving occlusion more effectively. In addition, the Average Confidence Score (ACS) of the fuzzy-based approach was always greater, reaching 99.21% in normal conditions and 95.60% in occluded conditions, against the lower ACS of the other approaches. These findings highlight the significant performance improvement that is realized by the integration of fuzzy logic into the ensemble approach, especially in enhancing robustness and reliability in real-world applications with occlusions. The Fuzzy-Based Ensemble Learning approach outperforms both standalone models, the Hybrid Approach and the other ensemble approaches in both standard and occluded conditions, demonstrating its improved ability to handle complex, real-world data variations, as shown by a comparison of these results with Tables 2, 3, 4 and 5.

**Table 5:** The performance comparison of different ensemble learning methods on better-performing models on the standard images and occluded bird object images test dataset.

Approach	Testing dataset with standard bird objects					Testing dataset with occluded bird objects				
	A (%)	*P* (%)	*R* (%)	FS (%)	ACS (%)	A (%)	*P* (%)	*R* (%)	FS (%)	ACS (%)
Simple Ensemble Learning	97.56	97.56	97.6	97.58	98.51	94.77	94.16	94.59	94.37	94.68
Fuzzy-Based Ensemble Learning	98.73	98.82	98.68	98.75	99.21	95.78	95.41	94.79	95.1	95.6
Random Forest Ensemble Learning	96.11	96.22	96.04	96.05	98.12	90.51	88.49	91.83	88.70	95.60
XG-Boost Ensemble Learning	89.68	91.22	89.55	89.71	98.21	75.95	73.32	79.02	71.13	95.6

A: Accuracy, P: Precision, R: Recall, FS: F1-Score, ACS: Average Confidence Score.

Random Forest and XGBoost Ensemble Learning tend to be better suited to structured/tabular data and fail to adequately utilize deep semantic features from CNN outputs. Their comparatively lower performance, especially under occluded conditions (e.g., XGBoost with 71.13% F1-score), shows their weakness in processing high-dimensional visual inputs and spatial variability. Simple Ensemble Learning, although superior to individual classifiers, handles all the images equally with equal weight contributions. In practice, however, some models receiving greater weight contributions will not provide optimal results for all input images, due to the model-specific capabilities or image complexity.

The primary cause of this enhancement was the adaptive aspect of fuzzy logic. In contrast to standard ensemble approaches that rely on fixed and model-independent weights, fuzzy logic dynamically allocates contribution weights to single models by considering confidence scores, feature importance, and contextual information from the input image. This rule-based, fine-grained decision-making allows the ensemble to assign greater importance to models that work better under certain conditions (e.g., low visibility, occlusion, or cluttered backgrounds), making the system more flexible and robust.

Additionally, the Average Confidence Score (ACS) is notably superior in the fuzzy-based approach (99.21% for regular images and 95.60% for occluded images), indicating a higher level of prediction confidence and reliability. This not only enhances performance measures but also renders the system more reliable in sensitive, real-world applications like wildlife monitoring and biodiversity conservation. Overall, the incorporation of fuzzy logic within the ensemble paradigm adds a context-sensitive, adaptive weighting strategy that effectively remedies the deficiencies of fixed-weight and decision-tree-based ensemble techniques. This results in more precise, confident, and stable forecasts, especially under difficult conditions such as occlusion and noise.

**Fig. 15:**
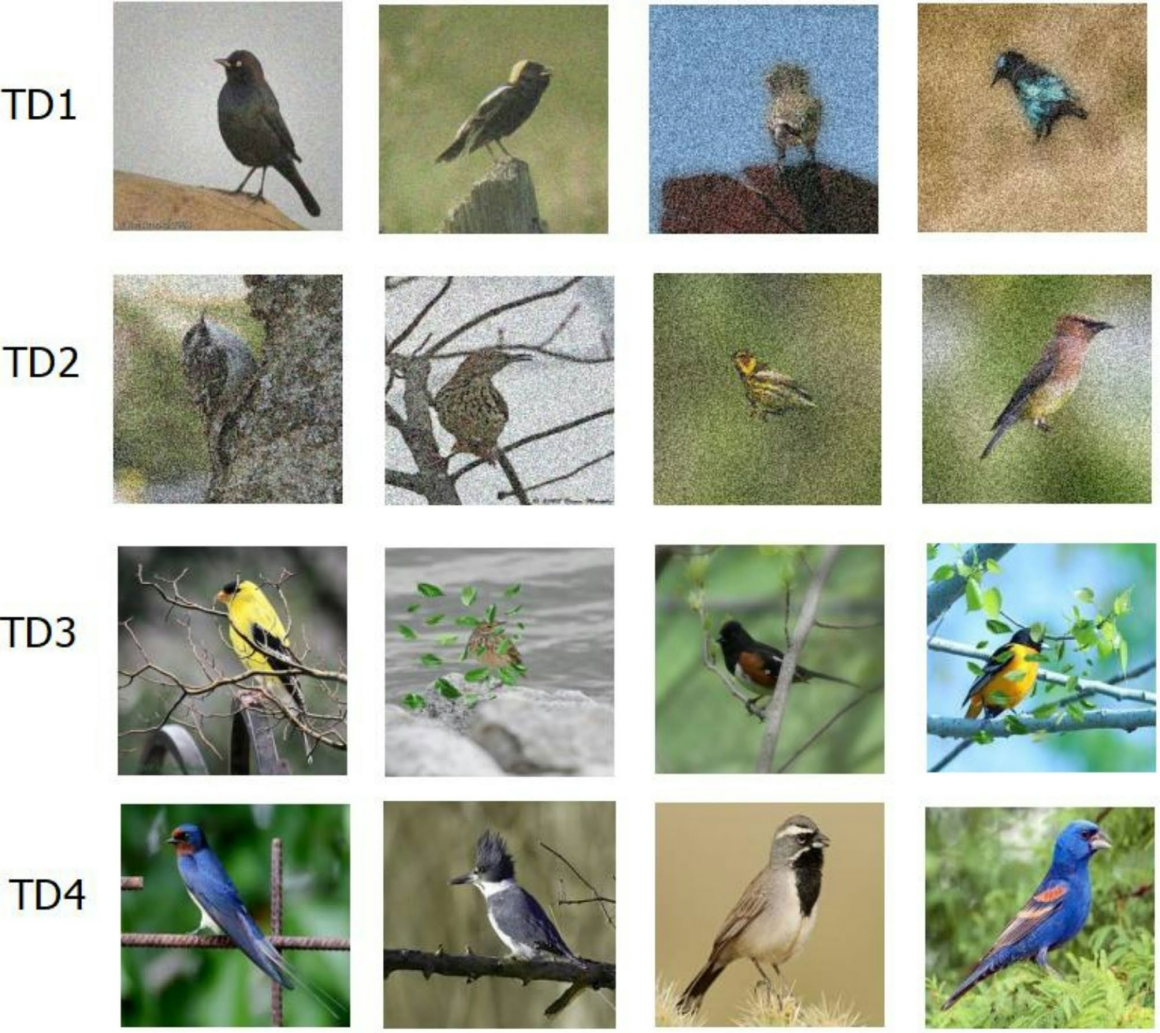
The sample images from the four challenge test datasets (TD1, TD2, TD3, and TD4).

To assess the effectiveness of the more effective ensemble learning techniques, we have developed four additional test datasets. To test the models’ resilience in various scenarios, these datasets—TD1, TD2, TD3, and TD4—each comprising 400 instances to simulate various real-world problems. TD1 and TD2, which depict environments with different noise levels, add 25–40% and 40–50% noise, respectively. To replicate natural occlusions, TD3 includes images with sporadic object insertions, including artificial leaves, shadows, and branches. With external data, TD4 provides a more broadly applicable test scenario since it uses entirely unseen images that were downloaded from the internet. The sample images from these four datasets are shown in Fig. 15.

These datasets assessed the performance of the Random Forest Ensemble, XGBoost Ensemble Learning, and Fuzzy-Based Ensemble Learning models, enabling a thorough evaluation of their generalization capacities. Table 6 displays the results from these new datasets, further illustrating how robust the ensemble learning techniques are, particularly when dealing with noise, occlusions, and hidden data fluctuations.

**Table 6:** Performance comparison of different ensemble approaches on the different complex test datasets.

Approach	TD1		TD2		TD3		TD4	
	A (%)	FS (%)	A (%)	FS (%)	A (%)	FS (%)	A (%)	FS (%)
Simple Ensemble Learning	97.27	97.29	97.01	96.89	90.30	91.72	96.96	96.88
Fuzzy-Based Ensemble Learning	98.25	98.27	97.97	97.85	90.91	92.28	98.03	98.13
Random Forest Ensemble Learning	96.36	96.12	90.00	88.80	90.91	90.81	93.11	93.05
XG-Boost Ensemble Learning	80.00	77.65	69.09	66.38	73.33	74.07	87.68	86.91

**TD1**: Testing Dataset with 25–40% Noise added, **TD2**: Testing Dataset with 40–50% Noise added, **TD3**: Test images with random object insertions such as synthetic leaves, shadows, and branches, designed to mimic occlusions in natural settings, **TD4**: Testing dataset containing completely unseen images downloaded from the internet.

According to Table 6, in TD1 (25–40% noise injected), Fuzzy-Based Ensemble Learning had the best accuracy (98.25%) and feature stability (98.27%), followed by Simple Ensemble Learning with 97.27% accuracy and 97.29% feature stability. Random Forest Ensemble Learning had slightly lower performance (96.36% accuracy, 96.12% feature stability), and XG-Boost Ensemble Learning trailed behind with 80.00% accuracy and 77.65% feature stability. In TD2 (40–50% added noise), Fuzzy-Based Ensemble Learning topped with 97.97% accuracy and 97.85% feature stability, and Simple Ensemble Learning again showed strong performance (97.01% accuracy, 96.89% feature stability). Random Forest suffered a performance loss (90.00% accuracy, 88.80% feature stability), and XG-Boost kept struggling (69.09% accuracy, 66.38% feature stability).

For TD3 (synthetic occluded images), Simple Ensemble Learning was marginally outperformed by Fuzzy-Based Ensemble Learning with 90.91% accuracy and 92.28% feature stability against 90.30% accuracy and 91.72% feature stability. Random Forest had similar accuracy (90.91%) but reduced stability (90.81%), while XG-Boost once more underperformed (73.33% accuracy, 74.07% feature stability). In TD4 (entirely unseen internet pictures), Fuzzy-Based Ensemble Learning continued its outstanding performance with 98.03% accuracy and 98.13% feature stability, while Simple Ensemble Learning ranked second (96.96% accuracy, 96.88% feature stability), Random Forest (93.11% accuracy, 93.05% feature stability), and XG-Boost (87.68% accuracy, 86.91% feature stability).

In general, Fuzzy-Based Ensemble Learning gave the most stable and robust performance on all difficult datasets, with both the highest accuracy and feature stability. Basic Ensemble Learning was close behind, while Random Forest gave moderate performance but was more sensitive to noise and novel data. XG-Boost Ensemble Learning gave the lowest values in all test scenarios.

To provide a reliable assessment and ensure a statistically sound analysis, 95% Confidence Intervals for accuracy (A) and feature score (FS) have been established based on evaluating the range in which actual metric values should fall. This ensures a reliable assessment and statistically sound analysis. The test dataset was enhanced using five transformation parameters within a structured evaluation framework: rotation (10–20 degrees), horizontal flip (0.1–0.5), vertical shift (0–0.1), and horizontal and vertical scale (0.01–0.1). After these augmentations were done using the parameters mentioned, the dataset was divided into five subsets for assessment. 5500 standard images initially used in the test dataset were augmented to 10,000 images, and each statistic run used 2000 images of the newly created test dataset. In the case of occluded images, the initial 1650 test images are augmented to 3000 images, which are divided into 600 for each assessment. Statistical testing was conducted using Ensemble Learning and Fuzzy Ensemble Learning with standard and occluded datasets, and assessment metrics are documented in Table 7 for five test runs. These findings were averaged to ensure that the performance evaluation would remain statistically sound and free from random variability between individual runs, and mean values, standard deviations, and 95% confidence intervals were computed and recorded in Table 7.

**Table 7:** Comparison of different statistical analyses between simple ensemble vs. fuzzy-based ensemble learning techniques on standard and occluded bird images datasets.

Approach		Testing metrics (Standard)		Testing metrics (Occluded)	
		A (%)	FS (%)	A (%)	FS (%)
Ensemble Learning	Run1-Set1	97.34	97.11	94.71	94.4
	Run2-Set2	96.11	96.19	94.67	94.19
	Run3-Set3	96.96	96.92	94.56	94.02
	Run4-Set4	96.57	96.57	93.51	93.49
	Run5-Set5	96.29	96.36	93.48	93.34
Mean ± Std. Dev		96.65 ± 0.50	96.63 ± 0.38	94.19 ± 0.63	93.89 ± 0.46
Mean ± 95%CI		96.65 ± 0.44 (96.21–97.09)	96.63 ± 0.33 (96.30–96.96)	94.19 ± 0.55 (93.64–94.74)	93.89 ± 0.40 (93.49–94.29)
Fuzzy-based Ensemble learning	Run1-Set1	98.19	98.21	95.47	95.08
	Run2-Set2	98.01	98.01	94.97	94.26
	Run3-Set3	97.51	97.59	94.61	94.22
	Run4-Set4	97.33	97.21	94.58	94.13
	Run5-Set5	97.24	97.09	94.21	94.12
Mean ± Std. Dev		97.66 ± 0.42	97.62 ± 0.49	94.77 ± 0.48	94.36 ± 0.41
Mean ± 95%CI		97.66 ± 0.37 (97.29–98.03)	97.62 ± 0.43 (97.19–98.05)	94.77 ± 0.42 (94.35–95.19)	94.36 ± 0.36 (94.00–94.72)
F-Statistic		11.74	12.82	5.42	5.35
p-value		0.0090	0.0072	0.0484	0.0494

A: Accuracy, FS: F1-Score.

From Table 7, Fuzzy Ensemble Learning regularly outperformed standard Ensemble Learning, with greater mean accuracy (97.66% vs. 96.65%) and FS (97.62% vs. 96.63%) on the standard dataset, as well as similar improvements in the occluded dataset (94.77% vs. 94.19% accuracy). The robustness of the Fuzzy Ensemble approach in successfully managing both standard and occluded scenarios is demonstrated by the steady outcomes indicated by confidence intervals. These results are further supported by a thorough p-value analysis, which shows that the performance difference between the two methods is statistically significant (*p* < 0.05), meaning that the observed improvements are unlikely to be the result of random chance. This further supports the efficacy and dependability of fuzzy ensemble learning in improving classification performance across various datasets. The F-statistic values presented in Table 7 (11.74 and 12.82 for standard data, 5.42 and 5.35 for occluded data) indicate a significant difference among the group means, corroborating that the differences in performance among ensemble and fuzzy ensemble learning algorithms are statistically significant.

Computational complexity analysis is critical while building a bird species classification system so that model performance can be efficient, particularly in constrained environments. It allows for real-time computation, facilitates scalability to large sets of data, and informs deployment to edge devices. It also allows for even trade-offs between model accuracy and efficiency, which helps in determining the right architectures to use for real-world applications. Table 8 shows performance and efficiency metrics for different bird species classification models/approaches. F1-Score (FS) and Accuracy (A) measure classification performance, while Multiply-Accumulate Operations (MAC, in GMAC) and Computational Parameters (CoP, in millions) reflect the computational complexity of the model. Average Inference Time (AIT, in milliseconds) quantifies the model’s speed per image, showing the balance between accuracy and computational efficiency.

**Table 8:** Computational complexity comparison between better-performing Stand-alone models and the ensemble model.

Approach/ model	A	FS	MAC	MAC	AIT
	(%)	(%)	(GMAC)	(Million)	(ms)
Densenet121	95.07	95.13	2.9	7.01	16.259
Densenet169	94.96	94.94	3.44	12.58	23.579
Resnet101	96.04	96.02	7.86	42.61	12.449
Simple Ensemble Learning	97.56	97.58	14.2	62.2	11.233
Fuzzy-Based Ensemble Learning	98.73	98.75	14.2	62.2	30.941

A - Accuracy, FS – F1-Score, MAC - Multiply-Accumulate Operations, CoP - Computational Parameters, AIT - Average Inference Time per Image.

Table 8 reveals that as model performance improves, computational requirements also increase in the proposed Fuzzy-Based Ensemble Learning approach. DenseNet121 achieves 95.07% accuracy with 7.01 GMACs and 2.9 million parameters, offering a relatively low computational cost. DenseNet169 has slightly lower performance at 94.96% accuracy, but requires more resources, with 12.58 GMACs and 3.44 million parameters. ResNet101 outperforms both with 96.04% accuracy and 96.02% F-score, but demands 42.61 GMACs and 7.86 million parameters, indicating a higher computational load. Simple Ensemble Learning further boosts performance to 97.56% and 97.58% F-score, but at a higher computational cost of 62.2 GMACs and 14.2 million parameters, with a still manageable inference time of 11.233 ms. The findings show that the fuzzy-based ensemble learning model demonstrates a significant trade-off between accuracy and computational complexity. While it achieves the highest accuracy (98.73%) and F-score (98.75%), it also requires 14.2 GMac MACs and 62.2 million parameters, leading to increased computational demands. Additionally, the inference time per image is longer (30.941 ms). These findings show a trade-off between efficiency and performance, where the accuracy improvements outweigh the higher computational expense.

The high computational cost and elevated inference time of the Fuzzy-Based Ensemble Learning approach, although achieving better accuracy, can potentially be problematic for real-time scenarios on low-power or edge devices. Future research can investigate techniques such as model pruning, quantization, and knowledge distillation to enhance deployability without particularly diminishing performance.

### Performance comparisons on the existing state-of-the-art research

Identifying fine-grained bird species remains challenging due to modest inter-class variances and substantial intra-class differences. Several recent publications have proposed unique deep-learning algorithms to address these complexities. Yi et al. (2023)^15^ proposed a modified YOLOv5-based bird classification model utilizing a part-based detection approach, where the classification is performed by inspecting specific regions of the bird rather than the entire image. Even though the authors do not refer to it as that, this approach is particularly suited to scenarios where just part of the bird can be seen, which is one form of occlusion. Their model’s 86.6% accuracy, however, indicates that it is limited in its ability to capture the broad range of features needed for accurate classification. A transfer learning method based on MobileNetV2^14^ had good accuracy (96.79%) on a small dataset classifying 20 bird species from South India, but its performance can degrade in complex scenarios where feature generalization is critical. A CNN model with skip connections was presented by Farman et al. (2023)^12^, which achieved 92% accuracy for a dataset of 20 species and 89.5% accuracy with VGG16. However, VGG16’s performance decreased to 60% for a bigger dataset of 525 species, indicating difficulties in scaling to different species. Although these studies provide significant contributions to the classification of bird species, one significant shortcoming is that they do not specifically address the difficulties associated with classifying partially visible birds. The outcome of the above existing works and our proposed works are presented in Table 9.

**Table 9:** Performance comparison of existing research techniques and the proposed approach.

Model/ approach Name	No. of classes	Type of objects	Performance metrics			
			Standard images		Occluded images	
			Accuracy	F1-Score	Accuracy	F1-Score
YOLOv5 + Res2Net-CBAM + CBAM ^15^	200	Standard (Considering Occulated)	NA	NA	86.6	65.45
MobileNetV2^14^	20	Standard	96.79	NA	NA	NA
CNN + Skip Connections^12^	20	Standard	94	94	NA	NA
Fuzzy- Based Ensemble Learning (Proposed Work)	55	Standard, Occulated	98.73	98.75	95.78	95.1

In contrast, our proposed fuzzy-based ensemble learning approach surpasses current existing methods by combining multiple deep-learning models, resulting in robust feature extraction and improved classification accuracy, as observed in Table 9. Our model performs well on occluded bird object images with 95.78% accuracy and 95.1% F1-score, and also attains 98.73% accuracy on standard bird object images. The fuzzy-based method dynamically modifies the limits of decisions, leading to improved resilience in complex scenarios.

### Ablation study

An ablation study is an approach for analyzing the contributions of different model components by gradually removing or changing them and observing how they affect performance. However, in the case of pre-trained models, an ablation study is not required on components like layers or connections, as models have highly efficient architectures and have already been optimized on large datasets like ImageNet. Instead, an ablation study was conducted on hyperparameters to choose the suitable candidates to increase the performance of the model, which was accomplished by freezing layers, optimizers, and learning rate (LR) since these are the important parameters affecting the model performance in bird species classification. We have performed the ablation study on the top-performing models in three levels, namely,

- Optimizers.
- Learning Rate (LR).
- Freezing layers.

Table 10 demonstrates that models trained with RMSProp at a learning rate of 1e-5 attain the highest accuracy and F1 scores, particularly for occluded images, and consistently outperform other configurations. When compared to models trained with ADAM or higher learning rates, with the RMSProp at a learning rate of 1e-5, DenseNet121 (95.07% Accuracy, 95.13% F1-score), DenseNet169 (94.96% Accuracy, 94.94% F1-score), and ResNet101V2 (96.04% Accuracy, 96.02% F1-score), perform better. The efficiency of our selected configuration is further supported by the fact that DenseNet121 with ADAM at 1e-3 drastically reduces its accuracy to 90.38%, whereas RMSProp at 1e-5 greatly increases it to 95.07%. Our choice is further supported by the fact that ResNet101V2 with ADAM at 1e-3 peaks at just 89.32%, while RMSProp at 1e-5 reaches 96.04%. These results support our selection of RMSProp with 1e-5 as the ideal configuration, guaranteeing enhanced robustness and greater generalization, particularly in challenging scenarios with occluded images. RMSProp dynamically adjusts the learning rate for every parameter. Along with the lower learning rate of 1e-5, it allows for small, steady weight updates, preventing divergence or instability and guaranteeing better convergence and generalization, particularly for complex data distributions like occluded bird images.

**Table 10:** Performance comparison of different optimizers and learning rate configurations on standard images and occluded images.

S. No.	Model Name	Ablation components		Standard bird objects		Occluded bird objects	
		Optimizer	Learning Rate	A (%)	FS (%)	A (%)	FS (%)
1	DenseNet121	ADAM	1e-3	90.38	90.39	81.01	77.37
2	DenseNet121	ADAM	1e-4	94.8	94.82	89.24	90.01
3	DenseNet121	ADAM	1e-5	93.3	93.41	86.08	83.54
4	DenseNet121	RMSProp	1e-3	75.35	76.58	60.57	60.24
5	DenseNet121	RMSProp	1e-4	81.25	81.47	70.89	69.78
6	DenseNet121	RMSProp	1e-5	95.07	95.13	91.77	91.17
7	DenseNet169	ADAM	1e-3	88.61	88.86	79.75	78.47
8	DenseNet169	ADAM	1e-4	94.65	94.71	89.24	88.75
9	DenseNet169	ADAM	1e-5	93.49	93.52	87.97	87.56
10	DenseNet169	RMSProp	1e-3	75.08	75.86	60.87	59.66
11	DenseNet169	RMSProp	1e-4	81.52	81.78	70.89	71.77
12	DenseNet169	RMSProp	1e-5	94.96	94.94	92.14	90.16
13	ResNet101V2	ADAM	1e-3	89.92	90.01	83.54	83.57
14	ResNet101V2	ADAM	1e-4	95.65	95.7	91.41	91.59
15	ResNet101V2	ADAM	1e-5	95.91	95.62	91.77	91.85
16	ResNet101V2	RMSProp	1e-3	74.58	75.91	61.87	61.42
17	ResNet101V2	RMSProp	1e-4	82.41	82.66	62.66	63.07
18	ResNet101V2	RMSProp	1e-5	96.04	96.02	91.41	91.59

A: Accuracy, FS: F1-Score.

The ablation study in Table 11 assesses how trainable layers affect model performance and demonstrates that fully trainable models (100%) perform better than partially trainable, where 50% of convolution layers are fine-tuned, and frozen models, where none of the convolution layers are fine-tuned. For Standard images, ResNet101V2 with 100% trainability earns the maximum accuracy (96.04%) and F1-Score score (96.02%), while for occluded images, it scores 91.41% accuracy and 91.59% F1-Score. Similarly, DenseNet121 (95.07% Accuracy, 95.13% F1-score for standard; 91.77% Accuracy, 91.17% F1-score for occluded) and DenseNet169 (94.96% Accuracy, 94.94% F1-score for standard; 92.14% Accuracy, 90.16% F1-score for occluded) exhibit optimal performance when completely trainable. It is confirmed that training all convolution layers improves classification Accuracy and F1-score, especially for occluded bird species where the prediction is complex, as there is a discernible decrease when layers are frozen. Allowing all layers to be trainable enables the model to learn richer hierarchical features, capturing fine-grained details essential for distinguishing similar bird species, whereas limiting trainability to 50% restricts feature refinement, leading to suboptimal representations and reduced classification performance.

**Table 11:** Performance comparison of trainable and frozen layers for standard images and occluded bird images.

S. No.	Model Name	Ablation components		Standard bird objects		Occluded bird objects	
		Trainable	Rate	A (%)	FS (%)	A (%)	FS (%)
1	DenseNet121	True	100%	95.07	95.13	91.77	91.17
2	DenseNet121	True	50%	92.22	92.36	89.67	89.12
3	DenseNet121	False	100%	75.87	76.67	61.39	61.46
4	DenseNet169	True	100%	94.96	94.94	92.14	90.16
5	DenseNet169	True	50%	91.87	91.13	90.34	89.78
6	DenseNet169	False	100%	74.76	75.48	62.18	62.45
7	ResNet101V2	True	100%	96.04	96.02	91.41	91.59
8	ResNet101V2	True	50%	93.45	93.23	89.32	89.07
9	ResNet101V2	False	100%	76.12	76.07	61.24	61.42

A: Accuracy, FS: F1-Score.

An ablation study is conducted for ensemble learning to evaluate its effectiveness in bird species classification. We examine the effect of weighing by trying varying distributions of weight to model contribution on the resulting prediction in simple ensemble learning.

**Table 12:** Impact of weight distribution on ensemble learning for bird species classification.

S.No	Ablation components			Standard bird objects		Occluded bird objects	
	DenseNet121	DenseNet169	ResNet101V2	A (%)	FS (%)	A (%)	FS (%)
1	Weight = 0.3	Weight = 0.3	Weight = 0.4	97.56	97.58	94.77	94.37
2	Weight = 0.2	Weight = 0.2	Weight = 0.6	96.88	96.84	93.41	92.89
3	Weight = 0.2	Weight = 0.3	Weight = 0.5	96.89	96.85	92.14	91.46
4	Weight = 0.3	Weight = 0.2	Weight = 0.5	96.96	96.92	93.41	92.72

A: Accuracy, FS: F1-Score.

Table 12 presents an ablation study that examines the effect of different weight distributions among various models in an ensemble learning framework. The findings show that classification performance is greatly improved by an optimal contribution balance. The effectiveness of this weighting technique is demonstrated by the best-performing configuration (0.3, 0.3, 0.4), which obtains the maximum accuracy (97.56%) and F1-Score (97.58%) for standard images, as well as 94.77% accuracy and 94.37% F1-Score for Occluded images. The most effective weighting configuration (0.3, 0.3, 0.4) assigns a higher weight to ResNet101V2, as it is the best-performing standalone model. While DenseNet121 and DenseNet169 receive equal lower weights since they provide approximately similar feature representations, since their pattern of feature extraction is preferably the same, other than the number of layers. This balanced yet performance-driven contribution improves overall classification resilience and accuracy, particularly in obstructed environments. These findings demonstrate that a fuzzy-based ensemble learning strategy is essential since adaptive weight assignment is crucial for maximizing model performance.

We also compare different fusion strategies, such as majority voting, mean voting, and weighted averaging, to figure out the best technique for combining the predictions. This study helps optimize the ensemble model for improved classification performance.

**Table 13:** Effectiveness of fusion strategies in ensemble learning for standard and occluded bird images.

S. No	Model name	Ablation components	Standard bird objects		Occluded bird objects	
		Voting	A (%)	FS (%)	A (%)	FS (%)
1	Ensemble	Majority Voting	96.42	96.44	91.77	88.15
2	Ensemble	Mean Voting	96.76	96.58	93.42	93.21
3	Ensemble	Weighted Averaging	97.56	97.58	94.77	94.37

A: Accuracy, FS: F1-Score.

In Table 13, a comparative study of fusion procedures for merging model predictions shows that Weighted Averaging performs noticeably better than Majority Voting. Weighted Averaging assigns different importance to each model’s prediction based on its reliability, ensuring that stronger models have a greater influence on the final decision, thereby enhancing classification performance. For Standard bird images, the proposed Weighted Averaging technique obtains the maximum accuracy (97.56%) and F1-Score (97.58%); for Occluded images, the results are 94.77% accuracy and 94.37% F1-Score. However, despite its effectiveness, majority voting yields slightly lower outcomes (96.42% Accuracy, 96.44% F1-score for standard; 91.77% Accuracy, 88.15% F1-score for occluded). Weighted averaging’s superior results confirm that it is used to leverage model confidence levels, which improves decision-making, especially in challenging occluded environments.

## Conclusion

This study suggested a CNN-based deep learning architecture for reliable bird species classification that is supplemented with a fuzzy-based ensemble learning framework. To increase classification accuracy in a variety of settings, the model successfully handles intricate visual obstacles such as occlusion, background clutter, and inter-class similarity. The Caltech-UCSD Birds-200-2011 and MyInfotech (Kaggle) datasets were utilized to pick the common 55 bird species that make up the dataset used in this study. Different methods of data augmentation, including rotation, scaling, and reflection, were used to encourage generalization. A hybrid multi-model method was used, combining traditional classifiers such as Random Forest, K-Nearest Neighbors (KNN), and Support Vector Machine (SVM) with discriminative characteristics from the top three performing CNN models.

This work’s use of fuzzy logic for determining the weights of each model’s contribution inside the ensemble is one of its main contributions. A more adaptable, robust, and interpretable decision-making process is achieved by using this method, which enables adaptive, context-aware weighting depending on the relative importance of each model’s attributes. Fuzzy logic can intelligently and dynamically adjust weights based on feature contributions, producing more resilient and adaptable ensemble models than set weighting methods employed in simple ensemble learning. On both conventional and occluded images, the fuzzy ensemble outperformed the simple ensemble by a margin of 1 to 1.5%. In terms of precision and recall, the proposed approach outperforms conventional CNN-based classification models, multi-model hybrid approaches, and straightforward ensemble learning with fixed weighting. Its significant accuracy of 95.78% for occluded images and 98.73% for normal images shows how well it performs in challenging real-time bird species classification tasks.

### Limitations and future works

- **Model Scope and Dataset Limitations**: Although the existing approach is effective, but specific to a subsample of bird species. More work will further increase the different bird species to enhance the generalizability across a broader range of different bird species. Transformer-based structures such as Vision Transformers (ViT) and Swin Transformers will also be investigated to deepen global feature learning and resilience against real-world deformations.
- **Static vs. Video-Based Classification**: Although the existing method emphasizes static image-based classification, there will be further research into enlarging the model towards video-based classification to enable real-time tracking and monitoring of bird species in natural environments. Such a development would broaden applicability in field-based conservation activities. Apart from that, fusion of multi-modal data, like bird sounds, GPS metadata, and environmental context, can enhance classification accuracy further, especially in poor conditions such as low visibility or uncertain visual cues.
- **Computational Complexity**: While our fuzzy-based ensemble model shows enhanced classification performance, the utilization of multiple heavyweight models adds to computational complexity, potentially making deployment problematic in resource-limited settings or for real-time systems. To overcome this, we intend to explore lightweight model architectures and utilize knowledge distillation methods. By transferring the acquired knowledge from heavyweight models into smaller, efficient student models, we aim to reduce these issues and facilitate more realistic deployment.

## Data Availability

The data that support the findings of this study are openly available, and the URL is mentioned below. The details are presented in reference numbers [21, 22]. https://www.kaggle.com/datasets/kedarsai/bird-species-classification-220-categories. https://www.kaggle.com/datasets/vinjamuripavan/bird-species.
